# Thioredoxin-80 is a product of alpha-secretase cleavage that inhibits amyloid-beta aggregation and is decreased in Alzheimer's disease brain

**DOI:** 10.1002/emmm.201201462

**Published:** 2012-08-30

**Authors:** Francisco Gil-Bea, Susanne Akterin, Torbjörn Persson, Laura Mateos, Anna Sandebring, Javier Avila-Cariño, Angel Gutierrez-Rodriguez, Erik Sundström, Arne Holmgren, Bengt Winblad, Angel Cedazo-Minguez

**Affiliations:** 1 Department of Neurobiology, KI-Alzheimer's Disease Research Center, Care Sciences and Society, Karolinska InstitutetHuddinge, Sweden; 2 Department of Cell and Molecular Biology, Karolinska InstitutetSolna, Sweden; 3 Cluster of Scientific Modeling, University of OviedoMieres, Spain; 4 Division of Neurodegeneration, Department of Neurobiology, Care Sciences and Society, Karolinska InstitutetHuddinge, Sweden; 5 Division of Biochemistry, Department of Medical Biochemistry and Biophysics, Karolinska InstitutetSolna, Sweden

**Keywords:** ADAM, Alzheimer's disease, amyloid-β, α-secretase, thioredoxin

## Abstract

Thioredoxin-1 (Trx1) is an endogenous dithiol reductant and antioxidant that was shown to be decreased in Alzheimer's disease (AD) neurons. A truncated form of Trx1, thioredoxin 80 (Trx80), was reported to be secreted from monocytes having cytokine activity. Here, we show that Trx80 is present in human brain in an aggregated form. Trx80 localizes mainly to neurons and is dramatically decreased in AD brains. Trx80 levels in cerebrospinal fluid (CSF) correlate with those of the classical AD biomarkers amyloid-β (Aβ) 1–42 and total tau. Moreover, Trx80 measurements in CSF discriminate between patients with stable mild cognitive impairment, prodomal AD and mild AD. We report that ADAM10 and 17, two α-secretases processing the Aβ precursor protein, are responsible for Trx80 generation. In contrast to the periphery, Trx80 has no pro-inflammatory effects in glia, either by itself or in combination with Aβ or apolipoprotein E. Instead, Trx80 inhibits Aβ(1–42) aggregation and protects against its toxicity. Thus, a reduction in Trx80 production would result in increased Aβ polymerization and enhanced neuronal vulnerability. Our data suggest that a deficit in Trx80 could participate in AD pathogenesis.

## INTRODUCTION

Thioredoxin-1 (Trx1) plays a central role in the control of cellular redox homeostasis and acts as an antioxidant (Arner & Holmgren, 2000). Trx1 has been shown to regulate apoptosis by inhibiting the apoptosis signal-regulating kinase-1 (ASK1; Saitoh et al, 1998). Trx1, directly or in association with other proteins like Trx1-interacting protein-2 (TBP-2), is known to play important roles in cell growth and survival, as well as in major metabolic diseases and cancer (Watanabe et al, 2010). Oxidative stress, apoptosis and inflammation are also key factors in neurodegenerative diseases, and there is an increasing interest in evaluating the role of Trx1 in such disorders (Lillig & Holmgren, 2007). We previously found that neuronal Trx1 is decreased in Alzheimer's disease (AD) brains and that Trx1 is oxidized by the β-amyloid (Aβ) peptide (Akterin et al, 2006). Aβ is the major constituent in the neuritic plaques seen in AD brains, and it is believed to be a driving force of the disease pathology (Hardy & Selkoe, 2002). Aβ results after sequential cleavage of amyloid precursor protein (APP) by β- and γ-secretases. Normally, a non-amyloidogenic pathway, involving α-secretases, is predominant in brain (Selkoe, 2001). In addition to Trx1, other members of the Trx1 family have been linked to AD, like some peroxiredoxins (Kim et al, 2001) and more recently Trx-reductase 2 (Cacho-Valadez et al, 2012). Together, these data emphasize the importance of Trx1 and related molecules in AD pathology.

Earlier studies on immune cells showed that Trx1 with 105 residues is cleaved to a 80–84 a.a. carboxy terminal-truncated protein, thioredoxin 80 (Trx80; Pekkari & Holmgren, 2004). Trx80 is found in plasma, secreted by monocytes (Pekkari et al, 2000). Indeed, Trx1 is also secreted, although the mechanism is not known since it is lacking a signal peptide. Trx80 levels in plasma are lower compared to Trx1, but there is no correlation between Trx80 and Trx1 levels (Pekkari et al, 2000). There are few reports showing Trx80 production in non-immune cells, such as cytotrophoblasts (Di Trapani et al, 1998) and rheumatoid synoviocytes (Lemarechal et al, 2007). Until now, the enzyme responsible for Trx80 production has not been identified. Also, there is no consensus on where the cleavage occurs. Studies in different cell models showed that Trx1 is either cleaved intracellularly and then secreted (Di Trapani et al, 1998; Lemarechal et al, 2007) or secreted and then cleaved extracellularly (Sahaf et al, 1997). It is known that Trx1 cleavage dramatically changes the properties of the protein, including a loss of oxido-reductase activity and the ability to be a disulphide substrate for thioredoxin-reductase (Pekkari et al, 2000). The main function described for Trx80 is to activate monocytes to secrete pro- and anti-inflammatory cytokines (Pekkari et al, 2005). Inflammation is an important component of neurodegenerative disorders, including AD. Activated glia produce pro-inflammatory cytokines that can be found in the cerebrospinal fluid (CSF) of AD patients (Cedazo-Minguez & Winblad, 2010). The present paper aimed to investigate the production of Trx80 in the brain and its contribution to AD pathology.

## RESULTS

### Trx80 is produced in brain and forms low-molecular-weight aggregates

**Figure 1:**
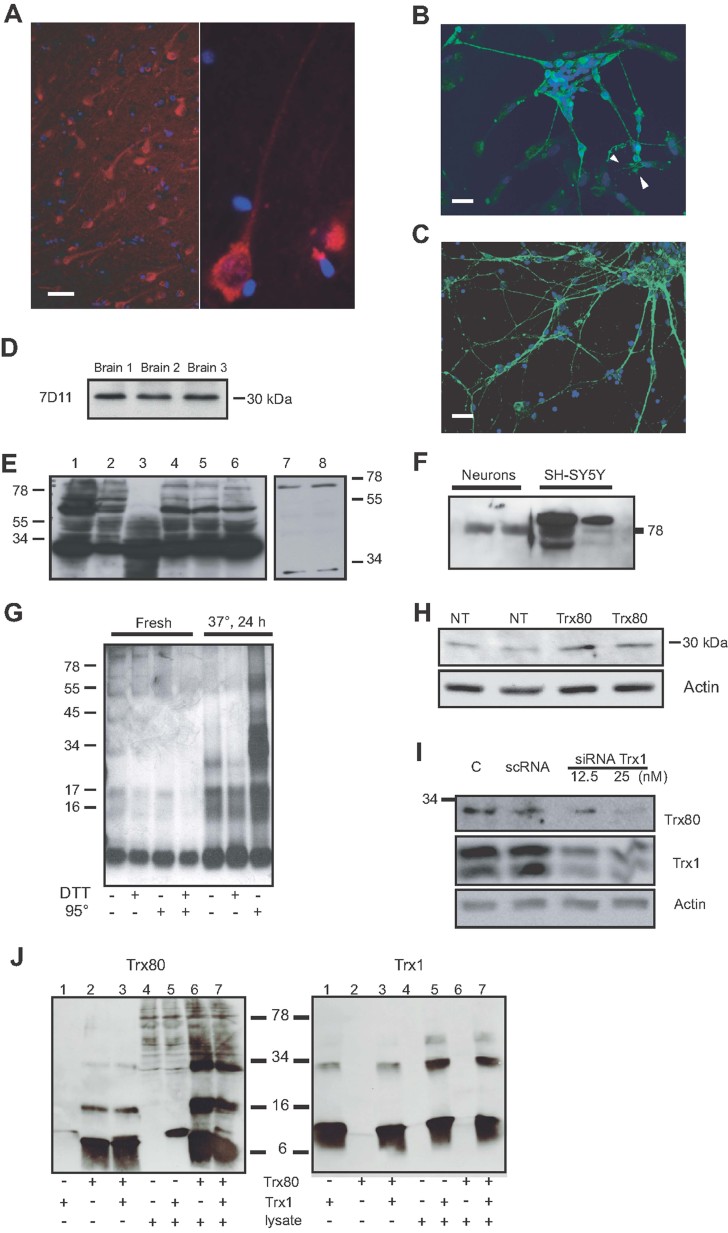
Trx80 is present in brain in an aggregated form **A–C.** Trx80 immunostaining in (**A**) human brain, (**B**) human mixed primary culture of neurons and astrocytes, (**C**) pure neuronal human primary culture. Trx80 is seen in neurons, particularly in bipolar and pyramidal neurons (right panel in **A**). Arrowheads in **B** indicate astrocytes. The pure neuronal cultures show Trx80 staining in soma and neuronal processes (**C**). Scale bars: 25 µm.**D.** Trx80 immunoblotting of samples from three control brains. The 7D11 antibody recognizes a band at approximately 30 kDa.**E.** Trx80 inmunoblotting of lysates from different cell types (1: Hela, 2: U2020, 3: U937, 4: SH-SY5Y, 5: SH-SY5Y overexpressing Trx1, 6: human primary neurons, 7–8: human primary glia).**F.** Trx80 is secreted and detected in the media from rat neuronal primary cultures and SH-SY5Y cells.**G.** Immunoblotting with the anti-Trx80 antibody 4H9. Recombinant Trx80 was incubated O/N at 37°C and compared to fresh Trx80. Samples were mixed with sample buffer ± DTT (10 mM) and left untreated or heated to 95°C.**H.** Transfected Trx80 also migrates as a band of approximately 30 kDa, as the endogenous Trx80. Panel shows two different clones.**I.** Reduction of Trx1 expression by siRNA resulted in decreased levels of the 30 kDa band detected by 7D11. Scrambled RNA (scRNA)-treated and vehicle-treated cells were used as controls.**J.** Lysates from SH-SY5Y cells were incubated (37°C, 24 h) with or without 2.4 µg of recombinant Trx80, Trx1 or both peptides together. As controls, Trx80 and Trx1 peptides were incubated at 37°C for 24 h in PBS. Blotting with anti-Trx80 (7D11) and anti-Trx1 abs is shown, respectively, in the left and right panels. Incubation of recombinant Trx80 with cell lysates resulted in more aggregated signals (left panel, lanes 2, 4 and 6). Addition of recombinant Trx1 alone or in combination with Trx80 did not change the aggregation pattern of Trx80 (left panel, lanes 3 *vs*. 2; 4 *vs*. 5; and 6 *vs*.7). The aggregation pattern of Trx1 was also changed by incubation with lysates, with the appearance of more dimers and trimers (right panel, lane 1 *vs*. 5). Addition of Trx80 did not change the Trx1 aggregation pattern (right panel, lanes 1 *vs*. 3 and 5 *vs*. 7).

Immunohistochemical studies of cortex and hippocampus from human brains showed that Trx80 immunoreactivity is mainly confined to pyramidal and bipolar neurons (Fig 1A). Trx80 immunoreactivity was confirmed in mixed (neuronal and glia) human primary cultures (Fig 1B). Using pure human neuronal cultures, we found that Trx80 is present in the soma and particularly in all neurites (Fig 1C). As seen in Fig 1D, the anti-Trx80 antibody 7D11 recognized a single band migrating at approximately 30 kDa in samples from temporal cortex of human brains. SDS–polyacrylamide gel electrophoresis (SDS–PAGE) analysis of lysates from several cell types, including human neurons and glia, showed that Trx80 immunoreactivity appears mainly as a single band of approximately 30 kDa and sometimes also as bands of 60 and/or 80 kDa (Fig 1E and Supporting Information Fig 1A). In conditioned media from primary human neurons and SH-SY5Y neuroblastoma cells, the 7D11 antibody recognized bands of approximately 80 kDa (Fig 1F).

The molecular weight (MW) of detected bands are not in agreement with the expected MW for Trx80 (10 kDa), which suggests the formation of aggregates. Immunoblotting of recombinant Trx80 with two different monoclonal antibodies able to recognize Trx80 (4H9 and 7D11) revealed the formation of aggregates after overnight (O/N) incubation at 37°C. Figure 1G shows the result achieved with the 4H9 antibody. As reported for other aggregated proteins such as α-synuclein (Sharon et al, 2001), heating to 95°C for 20 min prior O/N incubation increased Trx80 aggregation considerably. Addition of dithiothreitol (DTT; 10 mM) did not modify the aggregation pattern.

SDS–polyacrylamide gel electrophoresis (SDS–PAGE) analysis of SH-SY5Y cells transiently overexpressing Trx80 revealed that the increased amount of Trx80 migrated similarly as endogenous Trx80 from brain extracts, *i.e.* a band of 30 kDa (Fig 1H and Supporting Information Fig 1B). Reduction of the expression of Trx1 by siRNA (using 12.5 or 25 nM Trx1 siRNA), resulted in decreased levels of the 30 kDa band detected by anti-Trx80 antibodies (Fig 1I and Supporting Information Fig 1C). Moreover, recombinant Trx80 showed increased aggregation when incubated together with cell lysate (37°C, 24 h; Fig 1J), suggesting that Trx80 is prone to aggregate in a cellular milieu. This was also seen, although to a less extent, for recombinant Trx1 (Fig 1J). Co-incubation of both recombinant Trx80 and Trx1 with cell lysate did not change the aggregation pattern of the peptides compared to each peptide alone (Fig 1J).

**Figure 2:**
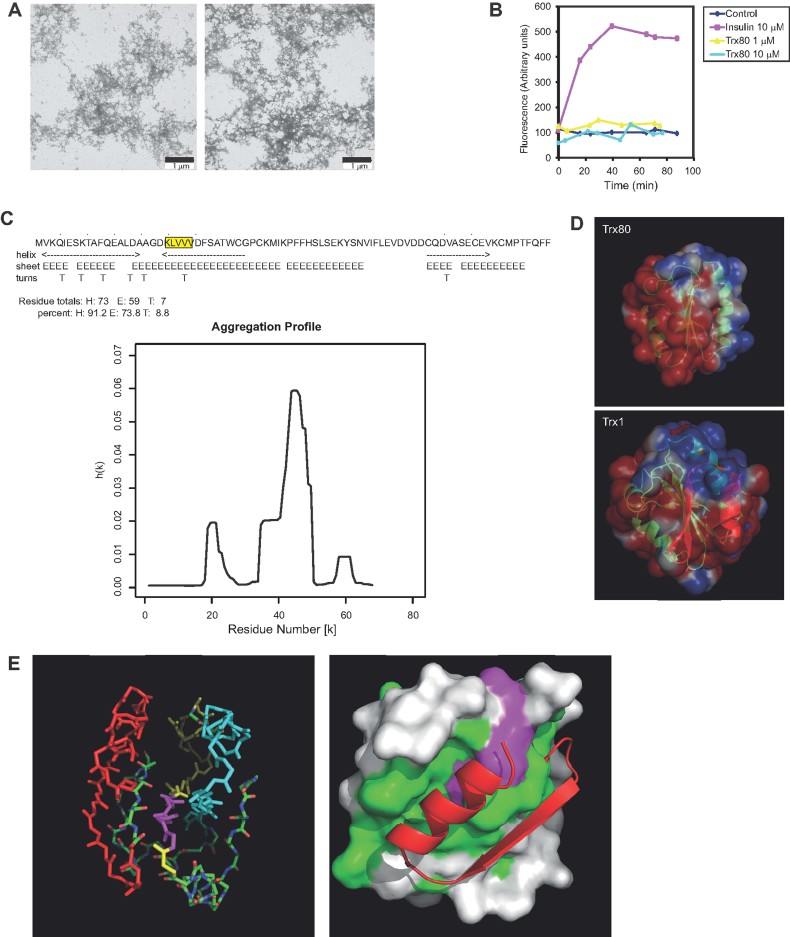
Aggregation of Trx80 Electron micrographs of recombinant Trx80 (10 µM) samples incubated O/N at 37°C in PBS (left) or heated to 95° prior to O/N incubation at 37°C (right). Formation of a diffuse network of aggregates was detected without structures resembling mature fibrils.This was confirmed by ThT assay. Insulin served as a positive control.Chou-Fasman plot and predicted aggregation profile of Trx80 revealed two major pro-aggregation areas, Trx80(20–37) and (47–60).Calculated surface of Trx80 using the X-ray diffraction-determined crystal structure for Trx1(1ERT). Red and blue areas show negative and positive potential, respectively.Trx1 backbone structure (using 1ERT coordinates) showing the key regions of the structure (left panel): in red, the differential segment with Trx80 and in yellow and cyan, the two possible aggregation zones from **D**. In the left panel, residues 21–25 (KLVVV) are shown in magenta. Trx80 calculated surface (right panel) showing the exposed hydrophobic patch (green) and residues 21–25 (magenta).

Electron microscopy revealed that O/N incubation of recombinant Trx80 (10 µM) in phosphate saline buffer (PBS) at 37°C induced the formation of a diffuse network of aggregates. No structures resembling mature fibrils were found (Fig 2A). Heating the samples to 95°C prior to O/N incubation at 37°C did not change the apparent structure of these aggregates (Fig 2a, right). The inability of Trx80 to form fibrils was confirmed by Thioflavin-T (ThT) fluorescence spectroscopy using insulin (10 µM) as control (Fig 2B). A prediction of aggregation (Trovato et al, 2007) profile of Trx80 revealed two major areas prone to aggregation: Trx80(20–37) and Trx80(40–60) (Fig 2C). Estimation of the Trx80 structure from the X-ray diffraction crystal structure of Trx1 [1ERT; (Weichsel et al, 1996)] revealed that Trx80 is a more hydrophobic molecule with exposure of the central β-sheets to the exterior (Fig 2D). PyMOL display of the protein backbone shows how the differential residues between Trx1 and Trx80 (residues 81–105 in red) shield the first predicted aggregation area (mainly a β-sheet that forms part of the hydrophobic core of Trx1) (Fig 2E, right). Figure 2E (left) displays the calculated surface of the Trx80 coloured green to show the location of the hydrophobic residues and, among them, the 21–25 residues KLVVV in magenta. The differential residues 81–105, depicted as a red ribbon, show how their cleavage exposes a big hydrophobic patch, which contains areas prone to aggregation (including the KLVVV residues).

### Trx80 levels are reduced in Alzheimer's disease

**Figure 3:**
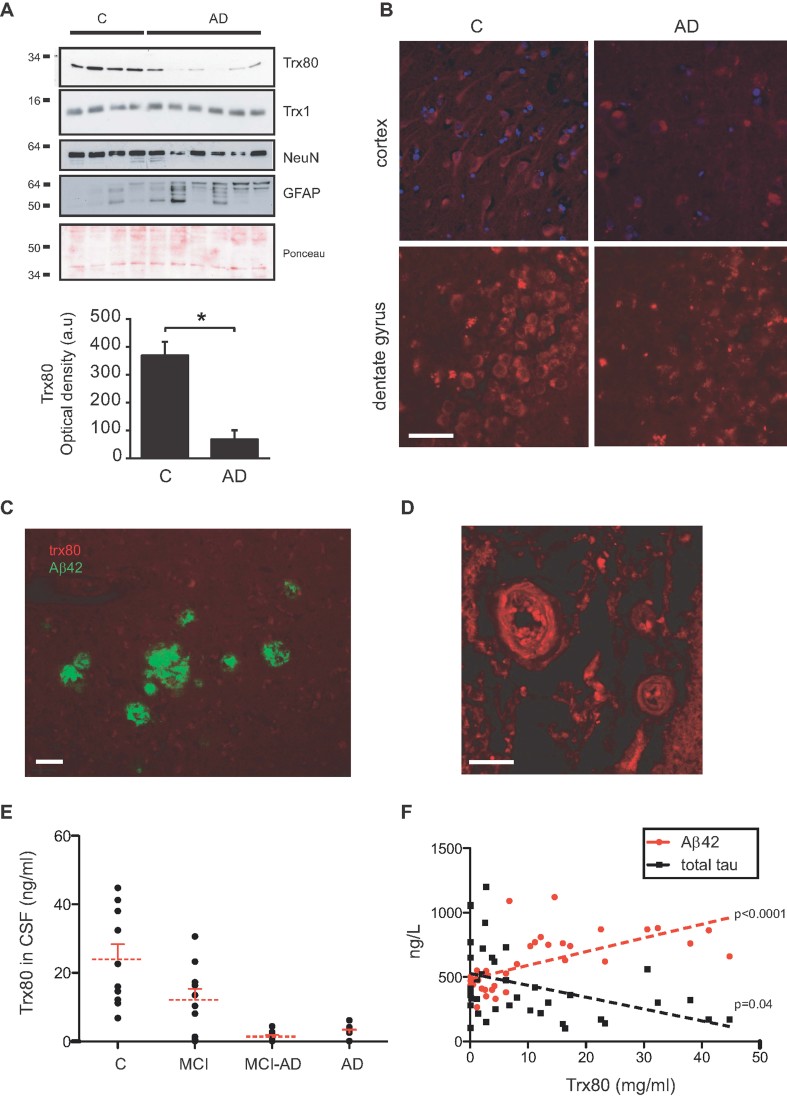
Reduced Trx80 levels in AD Immunoblotting of control and AD brain samples in human brain revealed a significant reduction of Trx80 in AD brains. No changes were found in Trx1 levels. As expected, NeuroN levels were reduced and GFAP levels increased in AD, reflecting neuronal loss and gliosis. Ponceau staining is shown as loading control. Histogram shows data expressed as optical density units and presented as means ± SEM (Mann–Whitney *U*-test; *p = 0.02).Trx80 staining is dramatically decreased in cerebral cortex and dentate gyrus of AD patients.Aβ(1–42) staining (green) does not co-localize with Trx80 staining (red) in senile plaques.One of the deep vessels with abundant Trx80 staining found in AD brains. Scale bars: 25 µm.Levels of Trx80 in CSF, measured by Sandwich ELISA, were reduced in samples from AD and MCI-progressing to AD patients compared to controls and MCI non-progressive patients (ANOVA, Bonferroni's multiple comparison test, *p* = 0.0001). A significant decrease was also found in non-progressive MCI samples compared to both controls and MCI-AD (*p* = 0.04).Levels of Trx80 in CSF correlated with Aβ42 levels [*r* = 0.6313; *p*(two-tailed) = 0.0001] and total tau [*r* = −0.4070; *p*(two-tailed) = 0.04].

Comparative analysis of samples from control and AD brains revealed a significant decrease of Trx80 immunoreactivity in AD (by 81.5% in five AD brain samples compared to four controls, *p* = 0.01; Fig 3A and Supporting Information Fig 1D). In AD brains, we saw a decrease in neuronal NeuN and an increase in glial fibrillary acidic protein (GFAP) (Fig 3A and Supporting Information Fig 1D) as indicators of neuronal loss and enhanced gliosis, respectively. Ponceau staining is shown as loading control (Fig 3A). Trx80 immunoreactivity was greatly reduced in both hippocampus (dentate gyrus) and cortex of AD brains compared with controls (Fig 3B and Supporting Information Fig 2A). Some staining was caused by autofluorescence from lipofuscin, heavily present in AD brains. Trx80 did not co-localize with Aβ fibril plaques (Fig 3C). AD samples had abundant Trx80 immunoreactivity in the wall of deep vessels in the white matter (Fig 3D).

To analyze Trx80 levels during AD progression, we quantified Trx80 in CSF from controls, from patients with stable mild cognitive impairment without progression to AD (MCI), MCI-progressive to AD (MCI-AD), or mild AD by a highly sensitive sandwich enzyme-linked immunosorbent assay (ELISA). Trx80-CSF levels were significantly decreased in both MCI-AD and AD compared to controls (Fig 3E; ANOVA, Bonferroni's multiple comparison test, *p* > 0.0001). A slight but significant decrease was also found in non-progressive MCI samples compared to controls and MCI-AD (*p* < 0.05). Levels of Trx80 correlated with Aβ(1–42) (*r* = 0.6313; *p* = 0.0001) and total tau (*r* = −0.4070; *p* = 0.04) levels (Fig 3F).

### Inhibition of ADAM10/17 suppresses Trx80 production

**Figure 4:**
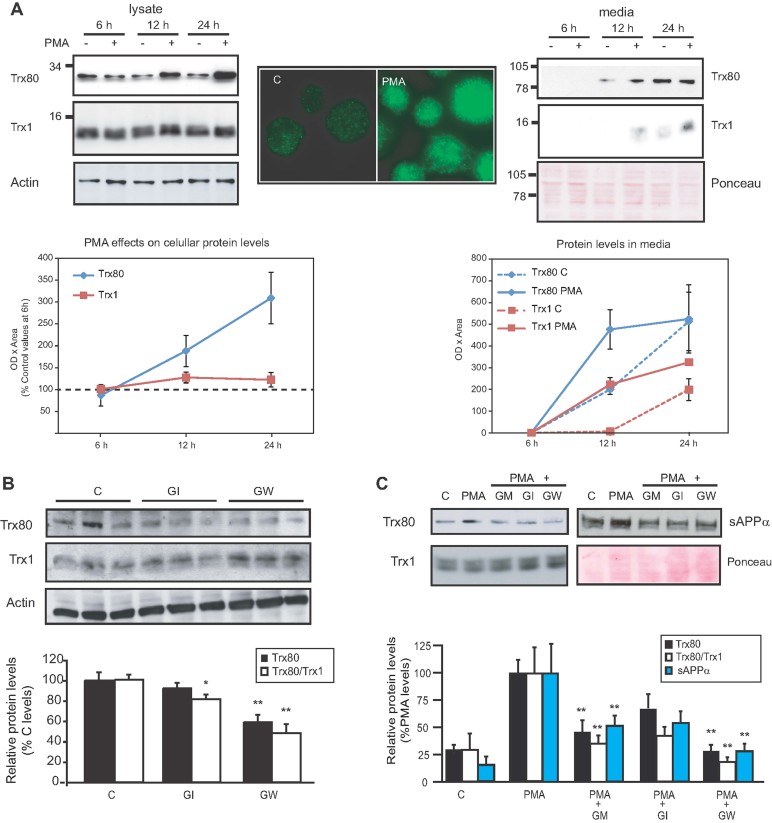
Trx80 is generated by α-secretases **A.** Trx80 levels both in cells (left and middle panels) and media (right panel) increase in response to PMA stimulation (50 ng/ml) of U937 cells. Trx1 secretion to the media is also increased by PMA (left panel) but the levels in lysates are not affected (right panel). Actin or Ponceau staining are shown as loading controls. Immunocytochemical pattern of Trx80 expression showed a dotted pattern that was increased after PMA treatment (middle panel). Graphs show quantifications (means ± SEM) of data.**B,C.** Effects of ADAM inhibitors on (**B**) basal or (**C**) PMA-stimulated (50 ng/ml) Trx80 and Trx1 production in SH-SY5Y cells. Bars represent mean values of four experiments ± SEM. Data are expressed as percentage of values for vehicle-treated cells (**B**) or PMA-stimulated levels (**C**). Analysis of the basal effects on Trx80/Trx1 ratio (**B** and **C**) and on PMA-stimulated sAPPα levels (**C**) are also shown. GM = GM6001 (10 µM); GI = GI254023X (10 µM); GW = GW280264X (10 µM). (ANOVA followed by Bonferroni's PLSD *post hoc* test; *p = 0.04 and **p *=* 0.009).

The regulatory mechanisms of Trx80 production from Trx1 as well as the enzyme responsible for the cleavage are unknown. As reported (Balcewicz-Sablinska et al, 1991), we found that phorbol 12-myristate 13-acetate (PMA; 50 ng/ml) increased intracellular levels of both Trx80 and Trx1 after 12 and 24 h in the monocyte cell line U937 (Fig 4A, left and middle panels; Supporting Information Fig 2B). At 12 and 24 h, Trx80 and Trx1 were also detected in conditioned media, the first as high MW aggregates (approx 80 kDa). Both, Trx80 and Trx1 secretion was stimulated by PMA (Fig 4A, right panel; Supporting Information Fig 2B–C).

Phorbolesters are known activators of α-secretase (Buxbaum et al, 1990). Lysine-directed secretases belonging to a disintegrin and metalloproteinase (ADAM) family, ADAM10 and 17, have been suggested as α-secretase candidates (Vincent & Checler, 2011), and α-secretase-mediated processing of APP is reduced in AD (Colciaghi et al, 2002). We hypothesized that α-secretases could be involved in Trx1 cleavage. To investigate this possibility, we used specific inhibitors of ADAM10 and 17 (Hundhausen et al, 2003). In SH-SY5Y cells, we found a reduction of Trx80 levels (of approx. 54%) after inhibition of both ADAM10/17 by GW280264X (10 µM). Inhibition of ADAM10 alone by GI254023X (10 µM) had a smaller effect. Accordingly, Trx1 levels were increased after inhibition of ADAM10/17 (Fig 4B). In U937 cells, the effects of both inhibitors were larger (59% and 32% reduction of Trx80 levels after inhibition of ADAM10 or ADAM10/17, respectively). PMA treatment (50 ng/ml, 24 h) enhanced Trx80 production (approximately four-fold of control levels; Fig 4C). This effect was partiality inhibited by the general metalloprotease inhibitor GM6001 (10 µM) and by ADAM10 inhibition. ADAM10/17 inhibition prevented completely the PMA effect (*p* = 0.01). ADAM inhibitors also reduced the PMA-mediated enhancement of secreted α-cleaved APP levels in a similar way to Trx80 (Fig 4C).

**Figure 5:**
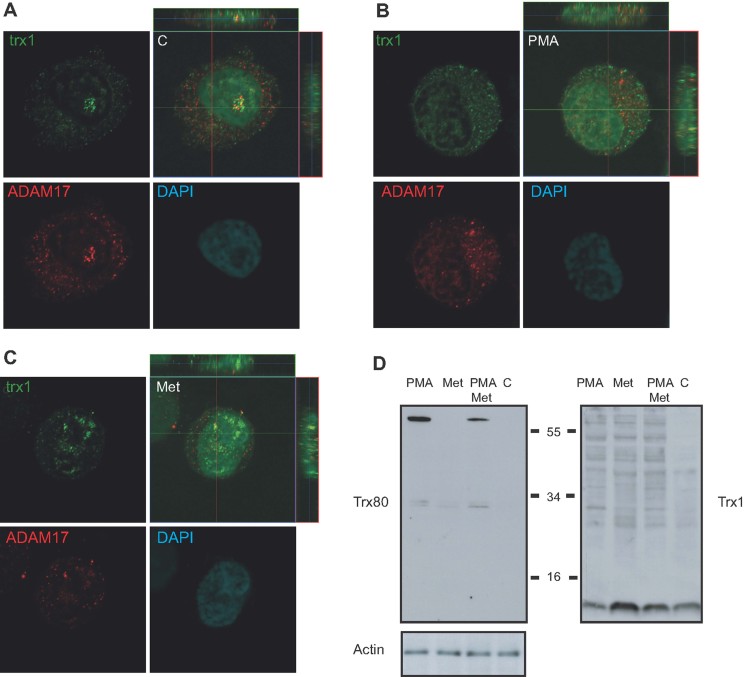
Co-localization of Trx1 and ADAM17 in cytoplasmic vesicles of U937 cells **A–C.** Co-localization of Trx1 (green) and ADAM17 (red) in (**A**) untreated (c), (**B**) PMA-treated (50 ng/ml) and (**C**) methylamine-treated (Met) (10 mM) U937 cells. Both single plane and z-stack images are shown. The co-staining (yellow) of Trx1 and ADAM17 was present in the cytosol, mainly in proximity of the nucleus. The dotted pattern of the staining suggests that Trx1 and ADAM17 could co-localize in vesicular compartments in the cytoplasm. Methylamine-treatment reduced the dotted pattern as well as the co-localization of Trx1 and ADAM17.**D.** Trx80 and Trx1 immunoblotting of lysates from U937 cells using 7D11 and anti-Trx1 antibodies, respectively. Pretreatment of cells with methylamine (10 mM, 5 h) reduced the PMA-mediated increase of Trx80.

We next looked for co-localization of ADAM17 and Trx1, as precursor of Trx80, using single plane and z-stack confocal imaging in U937 cells. As seen in Fig 5A, Trx1 and ADAM17 are co-localized to the cytoplasm, mainly in the proximity of the nucleus. No co-staining with DAPI was found, indicating a non-nuclear co-localization. PMA (50 ng/ml, 12 h) enhanced Trx1/ADAM17 interaction, which was extended to non-perynuclear areas (Fig 5B). The dotted staining pattern suggests that Trx1 and ADAM17 could co-localize in vesicular compartments in the cytoplasm. Methylamine that was shown to reduce the secretion of Trx1 by inhibiting the formation of vesicles involved in the non-classical secretory pathway (Rubartelli et al, 1992), reduced the amount of Trx1/ADAM17 co-localization (Fig 5C). In agreement with that, co-treatment with methylamine (10 mM, 12 h), reduced the PMA-mediated enhancement of Trx80 in U937 cells (Fig 5D). Co-localization of ADAM17 and Trx1 was also confirmed in SH-SY5Y cells (Supporting Information Fig 2D).

### Trx80 does not induce an inflammatory response in glia

Trx80 was reported to be a cytokine that stimulates monocytes to produce IL-12 and the anti-inflammatory IL-10 (Pekkari et al, 2001, 2005). We examined Trx80 effects in relation to two other players of AD pathology known to influence neuroinflammation, Aβ and apolipoprotein E (apoE). We used mixed cerebellar glial cultures (micro- and astroglia), with high expression of neuronal nitrogen oxide synthase (nNOS; Campese et al, 2007), as interrelations between both cell types are important to the final inflammatory effect. Nitrite release to the media was used as indicator of the inflammatory response (Privat et al, 1997). Cells were treated with fresh Trx80 (100 or 10 nM) alone or in combination with Aβ(1–42) (10 µM), Aβ(1–40) (10 µM), apoE3 (10 nM), or apoE4 (10 nM) or complexes (Aβ40/E3 or Aβ40/E4). Untreated cells or lipopolysaccharide (LPS) (100 ng/ml) treatment were used as negative and positive control, respectively.

**Figure 6:**
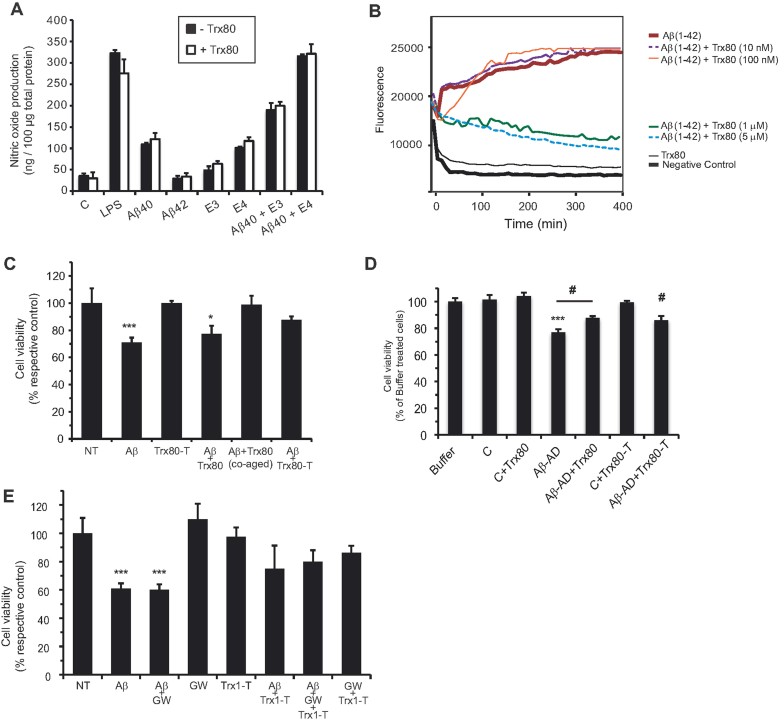
Trx80 is not pro-inflammatory in glia but inhibits Aβ(1–42) aggregation and toxicity Nitric oxide production was measured in mixed glial primary cultures from rat in response to Trx80 stimulation (10 nM) in combination with different inflammatory modulators. LPS = lipopolysaccharide (100 ng/ml); C = untreated cells; Aβ(1–42) (10 µM); Aβ(1–40) (10 µM); E3 = apoE3 (10 nM); E4 = apoE4 (10 nM). Data are expressed as ng of nitrites produced per µg of total protein. Bars represent mean values of three experiments ± SEM.Trx80 (1 and 5 µM) inhibited the fibrillar aggregation of Aβ(1–42) (20 µM) detected by fluorescence Thioflavin-T assay. Lower Trx80 concentrations were without effect.Aβ(1–42) (10 µM, 24 h) reduced cell viability in non-transfected (NT) and in Trx80 (100 nM)-treated SH-SY5Y cells. Aβ(1–42) was harmless when pre-incubated with Trx80 (100 nM) or added to Trx80-transfected (Trx80-T) cells. Data are expressed as percentage of the respective control and bars represent mean values of four experiments ± SEM (ANOVA followed by Bonferroni's PLSD *post hoc* test; ***p = 0.001, *p *=* 0.04).Aβ-rich fractions (1 µl) extracted from one AD brain (Aβ-AD) reduced cell viability of SH-SY5Y cells. The Aβ-AD effect was partially inhibited by addition of Trx80 (100pM) or by overexpression of Trx80 (Trx80T). Treatment with extracts from a plaque-free control brain (C) did not harm the cells. Data are expressed as percentage of values in buffer treated cells, and bars represent mean values of 9 experiments ± SEM (ANOVA followed by Bonferroni's PLSD *post hoc* test; ***p = 0.001 against controls; #*p* = 0.01 against Aβ-AD).Overexpression of Trx1 (Trx1-T) was partially protective against Aβ(1–42) (10 µM, 24 h) even under ADAM10/17 inhibition (GW280264X, 10 µM). Data from four experiments are shown as percents of MTT values from controls (untreated cells, NT) ± SEM (ANOVA followed by Bonferroni's PLSD *post hoc* test; ***p *=* 0.001).

As shown in Fig 6A, Trx80 alone (10 nM) did not induce changes in nitrites production. Aβ(1–40) and apoE4 induced moderate inflammatory responses. The effects were not seen with Aβ(1–42) or apoE3 treatments. Aβ(1–40)/apoE complexes, specially Aβ(1–40)/apoE4, induced significant increases in the accumulation of nitrites. The addition of Trx80 did not change the response to LPS, Aβ, apoE isoforms or Aβ/apoE complexes. Similar findings were obtained with 100 nM Trx80 and when Trx80 was incubated at 37°C O/N, to induce aggregates, prior to the addition to the cells (Supporting Information Fig 3A).

### Trx80 arrests Aβ aggregation and protects cells from Aβ toxicity

Polymerization of Aβ is critical for its toxicity, and inhibitors of Aβ aggregation have been proposed as potential treatment for AD. The 16–20 fragment of Aβ (KLVFF) was shown to be responsible for its aggregation (Tjernberg et al, 1996). Consequently, peptides binding to this fragment are able to inhibit Aβ polymerization (Tjernberg et al, 1997). From the two predicted pro-aggregation Trx80 regions, Trx80(21–25) (KLVVV) is exposed after cleavage of C-terminal Trx1 and shows important similarities to Aβ(16–20) (Fig 2). Therefore, binding between Trx80(21–25) and Aβ(16–20) could be possible. We evaluated the effect of Trx80 on Aβ(1–42) polymerization. Monomeric Aβ(1–42) (20 µM) aggregates into ThT reactive fibrils, an effect that was inhibited by co-incubation with monomeric Trx80 at concentrations of 1 and 5 µM (Fig 6B). Lower concentrations of Trx80 (10–100 nM) were without effect. As also described in Fig 2B, Trx80 alone did not show fibril formation (Fig 6B).

The effect of Trx80 on Aβ(1–42) toxicity was evaluated in SH-SY5Y cells (Fig 6C). Aβ(1–42) (10 µM), incubated O/N at 37°C to induce aggregates, significantly decreased cell viability at 24 h. This effect was not modified by coincubation with fresh Trx80 (1 µM). In contrast, Aβ(1–42) (10 µM) incubated together with Trx80 (1 µM) O/N was harmless to the cells. Moreover, pre-aggregated Aβ(1–42) (10 µM) was less toxic in cells transiently transfected to overexpress Trx80 (Trx80-T) (three different clones) (to 87.2 ± 2.8% of controls). We also tested the ability of Trx80 to protect against Aβ-rich extracts from AD brains. As seen in Fig 6D, incubation with Aβ-rich AD brain extracts reduced cell viability to 76.2% ± 2.3 compared with controls (extraction buffer-treated cells). This toxic effect was reduced by the addition (co-incubation) of a low concentration of recombinant Trx80 (100 pM) or by Trx80 overexpression (Trx80-T) (respectively to 87.7 ± 1.3 and 86.1 ± 2.9) (Fig 6D).

In an independent set of experiments, we determined the potential of Trx1 overexpression to protect against Aβ(1–42) toxicity in presence or absence of the ADAM10/17 inhibitior GW280264X. An approximately two-fold overexpression of Trx1 was partially protective against Aβ(1–42). This effect was not modified by GW280264X (Fig 6E).

## DISCUSSION

The reductant and antioxidant properties of Trx1 are lost when it is cleaved to Trx80 (Pekkari et al, 2003) but little is known about Trx80 function and how and in which tissues it is generated. The majority of studies on Trx80 were performed in immune cells where it acts as a cytokine for resting monocytes (Pekkari et al, 2000, 2005). Neurodegenerative disorders are often associated with oxidative stress and inflammatory processes. We previously reported a decrease in neuronal Trx1 levels in AD (Akterin et al, 2006). In the present study, we investigated the production of Trx80 in brain, the mechanisms of Trx80 generation in brain cells and whether Trx80 plays role in AD.

Using human brain samples and human primary cultures, we show that Trx80 is produced and polymerized into very stable aggregates of which molecular species migrating at approximately 30 kDa in SDS–PAGE are predominant in the brain. Furthermore, we show that Trx80 is secreted and forms larger aggregates in conditioned media from brain cells. Trx1 is mainly monomeric although it can form less active dimers (Powis & Montfort, 2001). Trx80 has a secondary structure similar to Trx1 but the removal of the C-terminal 24 residues increases the number of hydrophobic residues on the surface and, therefore, possible interactions (Pekkari et al, 2000). We also show that Trx1 and Trx80 do not aggregate with each other or interfere with their respective aggregations.

We found that Trx80 localization is mainly neuronal, predominant in the cytosol and abundant in axons and dendrites. Moreover, compared to normal brain tissue, Trx80 levels were drastically reduced in AD, even in areas with abundant inflammatory changes (*i.e.* surrounding the amyloid plaques). Trx80 levels were also drastically reduced in CSF from mild AD and MCI-progressive prodomal AD patients. Trx80 measurements in CSF clearly differentiate MCI converters from non-converters to AD, suggesting that Trx80 deficiency could be a specific feature of the disease. Trx80 showed a positive correlation with Aβ(1–42) and negative with total tau levels in CSF. Although the potential of CSF-Trx80 as AD biomarker could be suggested from our results, this matter should be properly investigated in a large sample cohort in which other neurodegenerative diseases should be included.

The function of Trx80 in brain cells seems to be different from what was described in immune cells, *i.e.* to stimulate a pro-inflammatory response (Pekkari et al, 2001). Treatment of rat primary glial cultures with recombinant Trx80 did not induce nNOS activity, either alone or in combination with Aβ or apoE. As previously reported (Chiarini et al, 2005; Colton et al, 2002; Takata et al, 2003), we found that Aβ(1–40) and apoEs (E4 > E3) induced a moderate increase in nNOS activity. On the other hand, and as also reported, Aβ(1–42) was without effect (Takata et al, 2003). Aβ(1–40)/apoE complexes [specially Aβ(1–40)/apoE4] triggered a more potent inflammatory effect. None of the responses seen with Aβ, apoEs or Aβ/apoE complexes were modified by the addition of Trx80, further supporting the idea that Trx80 does not have a pro-inflammatory effect on glial cells. Nitric oxide (NO) production can be related to arginine uptake, as arginine is the only substrate for all NOS isoforms (Alderton et al, 2001). Thus, it is possible that arginine uptake in glia is enhanced by apoE4 compared to apoE3 (Colton et al, 2002; Czapiga & Colton, 2003). The isoform-specific differences between Aβ(1–40)/apoE3 and Aβ(1–40)/apoE4 complexes constitute a interesting finding. The synergistic action of Aβ(1–40) and apoE4 on nNOS activity is likely to contribute to higher levels of oxidative and nitrosative stress seen in the brains of apoE4 carriers (Colton et al, 2002).

A reduction of its precursor (Trx1) levels in neurons (Akterin et al, 2006) does not entirely explain the Trx80 decrease seen in AD. Most likely, a downregulation of the enzyme (or enzymes) that cleave Trx1 to Trx80 also occurs. Pekkari and Holmgren (2004) suggested that Trx1 is cleaved by an inducible protease but its identity has so far been unknown. The protease involved in the synthesis of Trx80 should fulfill the following three criteria: (i) cleaves at lysine; (ii) is activated by phorbolesters and (iii) its activity is reduced in AD. We thought that α-secretase could be a suitable candidate. Using specific inhibitors of ADAM10 and 17, two enzymes with α-secretase activity (Vincent & Checler, 2011), we found that simultaneous inhibition of ADAM10 and 17 led to reduced basal and PMA-stimulated production of Trx80 in two different cell types, U937 and SH-SY5Y. Inhibition of ADAM10 alone had a reduced effect. ADAM10 has been shown to be responsible for both constitutive and PKC-regulated α-secretase activities, while ADAM17 appears to be mainly involved in the last one (Vincent & Checler, 2011). As PKC activity is greatly increased under PMA treatment, this would result in activation of both ADAM10 and 17. Under basal conditions, α-secretases are regulated by basal phosphorylation, and it is likely that several enzymes are contributing (including PKC). GI254023X is a specific inhibitor for ADAM10 (constitutive and induced α-secretase), while GW280264X inhibits both ADAM10 and 17 (again both constitutive and induced α-secretase activities). Thus, under basal conditions (Fig 4B), we see a reduction of Trx80 by both inhibitors, with GW280264X being more efficient, probably due to a more efficient inhibition of basal PKC-mediated α-secretase activity. Under PKC-stimulated conditions (PMA treatment, Fig 4C), GW280264X is more efficient than GI254023X in reducing Trx80 production. This is expected, as the combinatory effect of ADAM10 and ADAM17 blockages would inhibit the PKC inducible α-secretase more efficiently. Thus, our data strongly suggest that α-secretases are involved in the cleavage of Trx1 to generate Trx80. We show, as previously reported (Skovronsky et al, 2001), that levels of ADAM17 are not changed in AD. Accordingly, the decrease in Trx80 levels seen in AD would be a consequence of two co-operating factors: lower levels of its precursor (Trx1) (Akterin et al, 2006) and lower activity of the cleaving enzyme (α-secretase) (Tyler et al, 2002).

From the staining patterns of Trx1 and ADAM17, colocalization appears to occur in vesicles. Trx1 is secreted by a non-classical pathway that is still not identified but involves vesicular elements containing both cytosolic and membranous proteins (Rubartelli et al, 1992). Treatment of cells with methylamine has been shown to block the formation of endosomal vesicles, the secretion of Trx1 (Rubartelli et al, 1992), and other proteins secreted through a non-classical pathway (Mignatti et al, 1992; Nishihara et al, 2001). Using U937 cells, we showed that co-localization of Trx1 and ADAM17, as well as the levels of Trx80, were reduced by methylamine. PMA has the opposite effect on the cells, increasing the colocalization of Trx1 and ADAM17 within what appears to be cytosolic vesicles. Accordingly, PMA has previously been shown to activate secretion (Buccione et al, 1996) and to promote the internalization of ADAM17 from the plasma membrane (Doedens & Black, 2000). Consequently, we suggest that Trx1 and ADAM17 are recruited to a vesicular compartment destined for secretion and that their co-localization will enable the cleavage of Trx1 and the generation of Trx80.

From the aggregation profile of Trx80, we noted that the Trx80(21–25) (KLVVV) region could potentially bind to Aβ(16–20) (KLVFF), a critical region for Aβ polymerization and toxicity (Tjernberg et al, 1996). We demonstrated that Trx80 is able to inhibit Aβ(1–42) aggregation *in vitro*, as well as the toxicity of Aβ(1–42) in SH-SY5Y cells. The positive effects of Trx80 on Aβ(1–42)-induced toxicity were seen when Aβ(1–42) was preincubated with Trx80 prior to addition to the cells or when Trx80 was overexpressed. Trx80 was able to protect against both recombinant Aβ and purified Aβ-rich fractions from AD brains. As previously reported (Akterin et al, 2006), overexpression of Trx1 protected cells against Aβ toxicity. Our results suggest that this is independent of Trx80 generation since inhibition of ADAM10/17 did not alter this effect. Trx1 was shown to protect against Aβ through inhibition of ASK1 (Akterin et al, 2006). On the other hand, we see that Trx1 levels do not correlate with Trx80 levels, as reported previously (Pekkari et al, 2000). Together, these results indicate that improving Trx1 levels, activity or its cleavage to Trx80 would be beneficial to counteract toxic effects of Aβ. Since Trx80 lacks the dithiol-dependent antioxidative properties of Trx1, it is not possible to explain the protection by a reduction in oxidative stress. Our data rather suggest that inhibition of Aβ aggregation is the underlying mechanism of Trx80 protection. Thus, it is tempting to speculate that decreased Trx80 production seen in AD, caused by both reduction in neuronal Trx1 levels and in α-secretase activity, is likely to have a negative effect on the brain. A Trx80 deficit would result in increased Aβ polymerization and would make the neurons more vulnerable towards Aβ.

## MATERIALS AND METHODS

### Brain tissue

Brain material was obtained from the Brain Bank at Karolinska Institutet (Sweden) with approval by the Regional Ethical Review Board (Stockholm). For immunohistochemistry, four brains from patients with definite AD (Mirra et al, 1991; two males 75 and 86-year-old, two females75 and 86-year-old) and four controls (two females, 81 and 87 years old, and two males 66 and 79-year-old) were used. For immunoblotting, samples of the temporal cortex from six AD brains (three males and three females, 74–98 years old) and four controls (four males, 56–71 years old) were used. The brains had a post-mortem delay between 8 and 49 h.

### Immunohistochemical and immunocytochemistry

Immunohistochemistry and immunocytochemistry were performed as described previously (Akterin et al, 2006; Zheng et al, 2011). The primary antibodies used are described below in a specific section. All sections were treated simultaneously under the same conditions. For control staining, the primary antibody was omitted.

### Cell lines

Adherent cell lines were cultured as described previously (Cedazo-Minguez et al, 2001a), seeded at 50,000 cells/cm^2^ 2 days before the experiment. The non-adherent human monocytes U937 (Sundstrom & Nilsson, 1976) were cultured in RPMI 1640 with GlutaMAX supplemented with 10% fetal bovine serum (FBS) and seeded the same day as the experiment at 8 million cells per ∅10 cm dish. All cell culture supplies were purchased from Invitrogen Corporation (Sweden).

### DNA constructs and transfection

Based on the *TRX1* plasmid (Akterin et al, 2006), we amplified the Trx80 sequence using the forward primer 5′CGAATTCGCCACCATGGATTACAAGGATGACGACGATAA GATGGTGAAGCAGATCGAG-3′ and the reverse primer 5′-CGGATCCTTACTTAAA AAACTGGAATGTTGGCATGCATTTGACTTCAC-3. The forward primer introduced an *Eco*RI site followed by a Kozac sequence preceding the Flag epitope and the corresponding *TRX1* sequence. The reverse primer introduced a *Bam*HI site and a stop codon, which was placed after nucleotides that code for Lysine-80 (the last amino acid in Trx80). The amplified fragment was cloned into the *Eco*RI–*Bam*HI sites of the pIRESneo expression vector (Clontech, Germany) and the resulting plasmid was transformed into One Shot® TOP10 Chemically Competent *E. coli* (Invitrogen, USA) and selected with ampicillin (Invitrogen, USA). The resulting clone was confirmed by DNA sequencing (SeqLab, Germany). Transient transfections were performed using Lipofectamine™ 2000 (Invitrogen, USA) according to the manufacturer's instructions. Cells were grown in regular medium for 24 h, followed by a selective medium containing G418 geneticin (Invitrogen, USA).

### siRNA of Trx1

siRNA oligonucleotides and transfection reagent (DharmaFECT) were purchased from Thermo Scientific. The transfection was done according to the manufacturer's protocol. SH-SY5Y cells were cultivated in 6-well plates until 90% confluency was reached and transfected with two different siRNA concentrations, 12.5 and 25 nM. Twenty-five nanomolar of non-targeting siRNA was transfected as control. Cells were treated for 48 h before lysis.

### Human primary cultures

Embryonic human cortical tissue (6–11 weeks of gestation) was dissected after elective routine, first trimester abortions. Written informed consent was obtained from the pregnant women. The collection was approved by the Regional Ethical Review Board (Stockholm), in compliance with Swedish law. Neuronal cultures were grown in NeuroBasal medium with supplement of l-glutamine (0.5 mM) and B27 (2%). Mixed neuronal and glial cultures were grown in DMEM/F12/GlutaMax with N2 supplement (1%).

### Rat primary cultures

Hippocampal and cerebellar tissue from 16-day-old Sprague–Dawley rat embryos was homogenized in serum-free NeuroBasal medium with B27 supplement (2%). Cells from each embryo were seeded separately in dishes, pre-coated with poly-D-lysine MW 300,000 (0.17 mg/ml, Sigma–Aldrich, St. Louis, MO, USA) in PBS. Cells were grown for 2 weeks and the culture medium was changed every fourth day.

### Preparation of cell lysate and media for immunoblotting

U937 cells were collected and separated by centrifugation (1000×*g* for 10 min) at +4°C. For adherent cell lines and primary cultures media was collected and centrifuged (1000×*g* for 10 min) at +4°C. All cell types were lysed by applying lysis buffer (50 mM Tris–HCl pH 7.5, 150 mM NaCl, 1% Triton X-100, 2 mM EDTA, 2 mM EGTA), with protease inhibitor cocktail (Sigma–Aldrich, USA).

### Immunoblotting

Immunoblotting was performed as previously described (Akterin et al, 2006). Some immunoblots were stripped using Restore™ Western Blot Stripping buffer (Pierce, USA) and then re-blotted with other antibodies.

### Study population and CSF measurements

The patients included in the study (*n* = 40) were from the Geriatric University Clinic at the Karolinska University Hospital in Huddinge, Sweden: 10 had subjective cognitive impairment (SCI) and were considered as controls, 20 MCI, and 10 mild-AD. In the MCI group, 10 had progression-to-AD (MCI-AD) within 2 years time, and 10 remained stable (MCI). Description of samples and CSF extraction can be found in (Gil-Bea et al, 2010). Tau, P-Tau (P-Thr181) and Aβ1–42 were determined by ELISA (Gil-Bea et al, 2010; Innogenetics Belgium).

### Trx80 measurements by specific sandwich ELISA

Determination of Trx80 levels in CSF was performed with a sandwich ELISA as previously described (Pekkari et al, 2000). Standard samples of recombinant Trx80, coating antibodies (anti-Trx80 monoclonal mouse antibody 7D11) and detection antibodies (biotinylated goat polyclonal anti-Trx1) were from IMCO Corporation Ltd AB (Sweden). Standard dilutions of Trx80 (0.2–100 ng/ml) were prepared in blocking buffer, while CSF samples were undiluted. Fifty µl of standards or samples were added in duplicates and incubated O/N.

### Inhibition and stimulation of ADAM activity

Cells (U937 and SH-SY5Y) were treated with PMA (50 ng/ml), GM6001 (10 µM), GI254023X, (10 µM) and GW280264X (10 µM; Hundhausen et al, 2003), under serum-free conditions, for 24 h. Untreated cells were used as controls. The effect on Trx80 and Trx1 levels was analyzed by immunoblotting.

### Trx80 aggregation

Recombinant Trx1 (1 µg/7.1 µM) and Trx80 (1 µg/13.5 µM) were incubated O/N in PBS at 37°C. The recombinant protein was prepared as previously described (Pekkari et al, 2000). Equal volume of tricine gel sample buffer with or without DTT (10 mM) was added and then the samples were frozen before use. Half of the samples were boiled for 3 min at 95°C prior to SDS–PAGE. In the experiments with cell lysates, 100 µl of lysate from SH-SY5Y cells were incubated at 37°C for 24 h with or without 2.4 µg of Trx80 peptide. As control, Trx80 peptide was incubated at 37°C for 24 h in 100 µl PBS, pH 7.4. Samples were then analyzed with immunoblotting.

### Negative staining transmission electron microscopy

Trx80 (IMCO Corporation Ltd AB, Sweden) (10 µM in PBS pH. 7.4) was incubated O/N at 37°C. Some samples were boiled for 3 min at 95°C prior incubation O/N at 37°C. An aliquot of 4 µl was added to a grid coated with a Formvar supporting film coated with carbon for 5 min. The excess solution was soaked off by a filter paper, and the grid was stained with 0.5% uranyl acetate in water for 5 min and air-dried. Samples were visualized in a Tecnai FEI 10 electron microscope at 80 kV.

### Nitrite production

Rat primary glial cultures from cerebellum were grown for 2–3 weeks and then seeded in 48-well plates. At 80% confluence, the cells were incubated with serum-free DMEM medium overnight and the day after the media was removed and fresh serum-free DMEM was added containing the different treatments in the presence or absence of human recombinant Trx80 (10 nM); Aβ(1–40) (10 µM, Bachem AG, Switzerland), human recombinant apoE isoforms (E3 or E4) (10 nM, ReliaTech GmbH, Germany) or apoE/Aβ complexes. Aβ(1–40) was dissolved in serum free MEM media (pH 7.4) at a concentration of 10 µM and incubated for 24 h at 37°C prior to the addition to the cells. The apoE/Aβ complexes were made as previously described (Cedazo-Minguez et al, 2001a). NOS activity was measured by the accumulation of nitrites in the culture medium after 24 h, using the Griess reaction, as previously described (Privat et al, 1997). Data are expressed as ng nitrites/100 µg protein.

### Polymerization assay

Amyloid fibrillation of Trx80 was determined by ThT polymerization assay as described (Ivanova et al, 2009). Insulin was used as positive control. Samples were incubated at 37°C in triplicates.

Prior to the co-incubation study with Aβ(1–42) and Trx80, 1 mg Aβ(1–42) (Sigma–Aldrich, #A9810) was dissolved and prepared as described (Stine et al, 2003) in order to get an unaggregated preparation. Aβ(1–42) was diluted to 20 µM in 200 µl ThT buffer, with or without 5 µM Trx80 peptide. ThT buffer alone was used as negative control. The ThT fluorescence intensity was then recorded every 10 min with the same settings as described above.

**The paper explained**

PROBLEM:

Alzheimer's disease (AD) is the most prevalent of the dementias and a major public health problem. Diagnostic accuracy is illusive at the earliest stages and effective treatments are unconfirmed. How and by what mechanisms rare genetic mutations lead to AD is largely known, but we lack such understanding of the much more common sporadic forms of AD. Understanding contributing factors would likely yield valuable insights about mechanisms and lead to new therapeutic candidates as well as improvements in early diagnosis.

RESULTS:

We report that the endogenous antioxidant Trx1 is cleaved to thioredoxin 80 (Trx80) by α-secretases ADAM10 and 17. Trx80 localizes mainly as an aggregate in neurons and is dramatically reduced in AD brains. The decrease of Trx80 is detectable in CSF at early AD phases. Our measurements of Trx80 in CSF discriminated patients with stable mild cognitive impairment from prodomal AD and mild AD patients. We also demonstrated that Trx80 does inhibit amyloid-β aggregation (a critical issue in AD pathogenesis) and protects cells against amyloid-β toxicity.

IMPACT:

Our results strongly suggest that decreases in Trx80 in brain has a contributing role in AD pathogenesis and progression. Trx80 deficits would result in increased Aβ polymerization that, consequently, leaves neurons more vulnerable to Aβ. The search for inhibitors of Aβ aggregation is an important subject in AD, not only because of therapeutic implications, but also for diagnostic purposes. Since Trx80 measurements in CSF clearly differentiate MCI converters from non-converters to AD, the potential for CSF-Trx80 to be an AD diagnostic biomarker in early stage illness should be further investigated.

### Preparation of human brain samples for cell viability assay

Frozen human frontal cortex samples from one plaque-free neurologically healthy and one AD case were homogenized on ice in 1 × TBS (50 mM Tris–HCl, 0.15 M NaCl, pH 7.4) using a dounce homogenizer, and further ultracentrifuged at 175,000×*g* for 20 min. The pellets were dissolved and homogenized in 1% sodium dodecyl sulphate followed by ultracentrifugation at RT. This step was repeated four times to remove all the detergent soluble material. The remaining pellet was dissolved in 80% formic acid and immediately vortexed for 30 s. on maximum speed, followed by sonication in a 20°C water-bath. Samples were centrifuged at 25,000×*g* for 10 m. to clear the solution from any insoluble material. The supernatant was stored at −20°C. We determined the Aβ(1–42) concentration in the extracts by ELISA (human/rat β-amyloid 42 from Wako Chemicals GmbH, Neuss, Germany). Prior addition to the cells, extracts were neutralized to physiological pH by 2 M Tris–HCl (pH 9.0).

### Cell viability assay

Cell viability was assessed by a modified version of the MTT assay (Cedazo-Minguez et al, 2001a). SH-SY5Y cells (non-transfected and Trx80 overexpressing) were cultured in 24-well plates and treated for 24 h with or without 10 µM Aβ(1–42) (Sigma–Aldrich, USA). In the experiments with brain extracts, the effects of 1 µl Aβ-rich samples from AD brain, yielding a final concentration of 100 pm Aβ(1–42), were compared with the same volume of control brain samples. Results were expressed as percentages of the values obtained from the appropriate controls.

### Antibodies

The following antibodies were used: For Trx80, 7D11 (1:1000 for immunobloting and 1:500 for inmunocytochemistry) and 4H9 (1:1000), both from IMCO Corp Ltd AB (Sweden). Anti-Trx1 (1:1000, O/N, RT for immunobloting; and 1:500 for inmunocytochemistry, 1:1000 for immunoblotting, and 1:500 for inmunocytochemistry) Anti-Aβ(1–42), a gift from Jan Näslund, for immunohistochemistry as described in (Zheng et al, 2011). 6E10 for detectings APPα, as described (Cedazo-Minguez et al, 2001b). Anti-actin (1:1000 from Sigma–Aldrich, Sweden.O/N, RT). Anti-ADAM17 (H170) (1:100, from Santa Cruz Biotechnology, USA).

### Statistical analysis

Analyses of differences were carried out by ANOVA followed by Bonferroni's PLSD *post hoc test* or by Mann–Whitney *U*-test. A value of *p* < 0.05 was considered statistically significant in all analyses. Partial correlations with age, gender as confounding variables were used to assess associations between levels of total tau or Aβ(1–42) with Trx80 in CSF samples.

For more detailed Materials and Methods see the Supporting Information.

## Supplementary material

Detailed facts of importance to specialist readers are published as ”Supporting Information”. Such documents are peer-reviewed, but not copy-edited or typeset. They are made available as submitted by the authors.
