# Supplementary material for: Thioredoxin-80 is a product of alpha-secretase cleavage that inhibits amyloid-beta aggregation and is decreased in Alzheimer's disease brain
Source: EMBO Mol Med. 2012 Aug 30;4(10):1097–111. doi: 10.1002/emmm.201201462 (PMC3491839; doi:10.1002/emmm.201201462)
Supplement: Supplementary file 1 [file emmm0004-1097-SD1.pdf]

EMBO
Molecular Medicine

Manuscript EMM-2012-01462

Thioredoxin-80 is a product of alpha-secretase cleavage that inhibits amyloid-beta aggregation and is decreased in Alzheimerís disease brain

Francisco Gil-Bea, Susanne Akterin, Mr. Torbjörn Persson, Laura Mateos, Anna Sandebring, Javier Avila-Cariño, Angel Gutierrez-Rodriguez, Erik Sundström, Arne Holmgren, Bengt Winblad and Angel Cedazo-Minguez

Corresponding author: Angel Cedazo-Minguez, Karolinska Institutet

---

Review timeline:

Submission date:	12 April 2012
Editorial Decision:	09 May 2012
Revision received:	05 July 2012
Editorial Decision:	06 July 2012
Revision received:	14 July 2012
Accepted:	17 July 2012

Transaction Report:

(Note: With the exception of the correction of typographical or spelling errors that could be a source of ambiguity, letters and reports are not edited. The original formatting of letters and referee reports may not be reflected in this compilation.)

1st Editorial Decision 09 May 2012

Thank you for the submission of your manuscript "Thioredoxin-80 is a product of alpha-secretase cleavage that inhibits amyloid-beta aggregation and is decreased in Alzheimer's disease brain" to EMBO Molecular Medicine. We have now heard back from the referees whom we asked to evaluate your manuscript. You will see that the referees find the topic of your manuscript potentially interesting. However, they also raise significant concerns on the study, which should be addressed in a major revision of the manuscript.

In particular, reviewer #1 highlights that, in addition to experimental concerns that were also raised by the other reviewers, that the link between Trx1 cleavage and protection against Abeta toxicity should be further investigated.

Given the balance of these evaluations, we feel that we can consider a revision of your manuscript if you can convincingly address the issues that have been raised within the space and time constraints outlined below.

Revised manuscripts should be submitted within three months of a request for revision. They will otherwise be treated as new submissions, unless arranged otherwise with the editor.

I look forward to seeing a revised form of your manuscript as soon as possible.

Yours sincerely,

Editor
EMBO Molecular Medicine

***** Reviewer's comments *****

Referee #1 (Comments on Novelty/Model System):

Inadequate for Fig. 6 see report. Additional experiments are needed to support the hypothesis. Inadequate also because of the use of Ab40 and not Ab42 in panel a (see report)

Referee #1 (Other Remarks):

The paper by Gil-Bea and colleagues studies the processing of Trx1 by α-secretase candidates and suggest that the derived product Trx80 could protect against Aβ aggregates-related toxicity. They also show that levels of this Trx1-derived fragment are lowered in AD brains. The paper is interesting but several major issues have to be considered before suitability for publication at EMM.

Major points

Fig. 1: Gels are cut and should be shown in their entirety. Full gels can be added in a supplemental figure. The anti-Trx80 antibody labels only a 3OkDa in the human brain cortex, and appears reduced in AD brain. If only one band is also detected in pathological brain, what is the relevance of aggregates observed in cell lysates?
The panel j is not convincing. The difference is not obvious and difficult to estimate in absence of quantification of overall immunoreactivity.

Fig.2: What is the evidence that endogenous aggregates display the same biophysical properties of recombinant one? In the case of Aβ oligomers for instance, the lack of toxicity of synthetic oligomers indeed suggests that oligomers (in general) cud display distinct properties when they are from synthetic, recombinant or tissue-prepared origin.

Fig.3: again show whole gel in panel a. While panel e and Methods refer to interesting various groups of pre-AD (MCI), MCI-AD and AD, only AD is indicated in panel a without any precision. Which AD group is it? Is Trx80 changing according to the disease state? This should be established in order to potentially correlateTrx80 in CSF and in brains. Is there any statistical difference between MCI-AD and AD in panel e?

Fig.4: There is no loading control protein in panel a and no quantification of data. Inhibitors of ADAM used in the study are not specific as claimed by authors. This perhaps explains the fact that both GI and GW lower Trx80 formation in both basal and PKC-regulated conditions. Authors do not underline this apparent discrepancy with previous data on distinct ADAM10 and 17 susceptibilities to PKC-mediated phosphorylation. It would be better to down-regulate these enzymes by siRNA approach. How do authors explain the fact that Trx1 is not increased by GW in panel b (right) while it is increased (as expected for a precursor-product relationship) in panel b left?
What are the two bands seen for APPα labeling?

Fig.6: this referee does not understand why authors used Aβ40 here! On the other hand, Aβ42 is used in panel b and c. This set of experiments should be done with Aβ42 that is more prone to aggregation.
In a more pathologically relevant context, authors should examine whether Trx80 expressing cells protect against toxicity harbored by aggregates prepared from AD-affected brains as in Walsh and Colleagues studies.

Another important point is that authors do not directly establish whether α-secretase blockade by inhibitors increase Aβ-related toxicity in Trx1 expressing cells. This would reinforce the view that α-secretase-mediated Trx80 production could interfere with trx80 function. In this paper, although authors show that Trx80 derives from α-secretase cleavage of Trx1 and is apparently protecting when used as a recombinant protein or in cells overexpressing it, no direct link between the two

events (i.e Trx1 cleavge by secretase) and protection against Ab toxicity is provided. Thus, authors should overexpress Trx1, assess the influence of this surexpression on Ab40/42-mediated toxicity and examine α-secretase inhibitors affect Trx1 specific phenotype.

Minor points
-p7: Phorbol esters are known activators of α-secretase... Authors cite one of their reference that is not focusing on the point while original and many previous works are omitted!

-Several references should perhaps be updated if possible!

Referee #2:

In this work, Gil-Bea et al have indicated the decrease of a fragment of thioredoxin 1 (Trx1) in the brain of Alzheimer's patients. This finding could facility the use of that fragment as a marker for the disease. In that way, the authors found that the changes in the level of the Trx1 fragment, in CSF, correlate with the changes found for other CSF markers like Aβ and tau. The work is sound but some points must be addressed.

Specific points:

- Trx1 plays a neuroprotective role because it is an antioxidant and an apoptosis inhibitor. The truncated form of Trx1, Trx80, lacks the neuroprotective role and it arises after cleaving of Trx1 by α secretases. Thus, it will be of interest to compare the levels of these α secretases (for example that of ADAM17) in AD and control samples (Western blot).
- In Figure 3a, a protein loading control is missing and Figure 3f lacks the parameters measured in the ordinate.
- Also, some further editing is needed for the references of Vicent B and Checler F where volume and pages numbers are missing or for that of Zheng et al where the page numbers are not indicated.

Referee #3:

1) Data are generally quite interesting, covering human sample to protein level.

2) There have been no previous publications related to Trx80 in the field of clinical and basic neurobiology.
Therefore it is highly recommended to show comparative data between Trx80 and variety of anti-oxidant or redox-active principles in direct relation to alzheimer disease.

3) Minor Comments
3-1 Figure 3B As the background level in this Figure is quite different from the data in oher panels, suitable careful correction is recommended.
3-2 Figure 5 This U937 Data using non-neuronal cell line cells should be supported by any suitable neuro_endocrine cell types.
3-3 Figure 6 Additional data is needed to show dose dependency.

4) Suitable reference and discussion on the target molecueles of thioredoxin family proteind namely TBP2/TXNIP/VDUP1, Ref-1/APEX etc may improve the quality of this paper more relevant in the specific field of clinical and molecular neurodegenerative diseases.

5) It is critically important to discuss the relationshhip between Trx80 and Trx-1, including the possibility of the reduction of Trx80 dimer by Trx-1.

We would like to thank the editorial board for this opportunity to submit our revised manuscript on "Thioredoxin-80 is a product of alpha-secretase cleavage that inhibits amyloid-beta aggregation and is decreased in Alzheimer's disease brain". We thank the reviewers for the acknowledgement that the results are interesting and novel. We have considered all their comments and have modified the manuscript where appropriate. In the revised manuscript we have improved presentation or added new data to address these concerns. Please find bellow a point to point description of the changes:

REFEREE: 1

Fig. 1: Gels are cut and should be shown in their entirety. Full gels can be added in a supplemental figure. The anti-Trx80 antibody labels only a 30kDa in the human brain cortex, and appears reduced in AD brain.

Response: We shown now the entire gel scans of cut susceptible figures (1F, 1H and 1I) as supplementary (Supp Fig 1). Please note that Suppl. 1D shows the full gel of figure 3A, which, as figure 1D, shows Trx80 immunoreactivity in brain samples.

If only one band is also detected in pathological brain, what is the relevance of aggregates observed in cell lysates?

This is a difficult question. The functional relevance of the conformational versatility of aggregating proteins is an open question in general, but also in many neurodegenerative disorders, including AD (b-amyloid) and PD (a-synuclein). Aggregation of Trx80 was not reported before. In addition to WB of cell lysates, we also show that Trx80 form small diffuse aggregates by EM (Figure 2). Trx80 appears majoritary as a approx 30 kDa band in control brains, AD brains and cell lysates. Our hypothesis, as discussed in the paper, is that Trx80 aggregates could be the functional form of Trx80.
Because both human Trx1 and Trx80 have five cysteines, they can be modified under oxidative or nitrosative stress in different ways, affecting both the aggregation state and activity. This has been shown for the precursor (Trx1) were stable dimmers and aggregates have been described (Hashemy and Holmgren, 2008). Our results for Trx80 show that controls and AD samples do not present differences in aggregation but in levels. Indeed, much more experiments are required to understand the relationship between structure and function of aggregated proteins, also Trx80.

The panel j is not convincing. The difference is not obvious and difficult to estimate in absence of quantification of overall immunoreactivity.

Response: This figure has been changed. In the new experiments, we have used less concentration of recombinant Trx80 and incubated for longer time with the lysate. In addition, as response to Referee#2, we added additional groups including Trx1 and Trx1+Trx80, to investigate possible interactions in the aggregation of these two molecules. A blot for Trx1 is also included. Accordingly, Figure legend and Material and method sections have been changed.

Fig.3: again show whole gel in panel a.

Response: The whole gel was shown in the previous version in supplementary data (Figure B). In this new version it is in Supp Fig 1D. Images of loading controls have been added.

While panel e and Methods refer to interesting various groups of pre-AD (MCI), MCI-AD and AD, only AD is indicated in panel a without any precision. Which AD group is it? Is Trx80 changing according to the disease state? This should be established in order to potentially correlateTrx80 in CSF and in brains. Is there any statistical difference between MCI-AD and AD in panel e?

Response: Figure 3a shows Control and AD brains, but not brains of MCI patients. Indeed it is extremely difficult to find brain from individuals with MCI, since most likely death occurs with a

fully developed AD. To address the question of Trx80 changes along AD progression we used CSF samples (Figure 3E). There is not statistical significance between MCI-AD (early prodomal AD) and AD groups, which, as discussed in the manus, strongly indicate a decrease of Trx80 in early states of AD. We apologize for the lack of clarity in presentation in our previous version.

Fig.4: There is no loading control protein in panel a and no quantification of data. Inhibitors of ADAM used in the study are not specific as claimed by authors. This perhaps explains the fact that both GI and GW lower Trx80 formation in both basal and PKC-regulated conditions. Authors do not underline this apparent discrepancy with previous data on distinct ADAM10 and 17 susceptibilities to PKC-mediated phosphorylation. It would be better to down-regulate these enzymes by siRNA approach. How do authors explain the fact that Trx1 is not increased by GW in panel b (right) while it is increased (as expected for a precursor-product relationship) in panel b left?
What are the two bands seen for APPα labeling?
The images shown are representative of how many trials?

<u>Response:</u> Loading controls have been now added to the Figure, as well as the quantification of data resulting from 4 independent experiments.

- Previous figure 4b is now divided into 4B and 4C. Previous panel b-left (now 4B) show basal data (without PMA stimulation) while panel b-right (now 4C) shown data of treatments combining PMA± ADAM inhibitors. Following the referee advise, the different susceptibilities of ADAM 10 and 17 to PKC resulting in diverse constitutive or induced a-secretase activity are discussed in the revised version (see discussion). We apologize for the lack of clarity in the interpretation of these data, which we have improved in this version.

The referee suggests siRNA experiments of ADAMs as alternative approach to pharmacological inhibition. We agree that the results obtained are not easy to interpret. However, in our opinion, either siRNA experiments would not be of a straight forward interpretation. Even knockout mutants of the ADAM proteases do not completely abolish a-secretase activity. ADAM10-deficient mice are embryonic lethal due to defective Notch signaling, but embryonic fibroblasts from these mice maintain a-secretase activity (Hartmann et al., 2002). In ADAM17-knockout cells, phorbol ester induced secretion of sAPPa was abolished while the constitutive release of sAPPa was preserved (Buxbaum et al., 1998). Double ADAM10/17 KO would likely shown similar results as the GW inhibitor. Nevertheless, we believe that all the experiments show in Figure 4, obtained by three different strategies (co-imuno, pharmacological inhibition of ADAMs and blockage of the non classical secretory pathway) demonstrate the interaction of a-secretases and Trx1 to generate Trx80.

-We show now (4C) images (both Trx80 and Trx1) from other set of experiments, where appears a moderate increase of Trx1 after ADAM inhibitors. As in Figure 4B, quantification of Trx80/Trx1 ratios are shown for figure 4C.

- The two bands seen for sAPPa are commonly seen when using 6E10 abs (for example see Fig 3 C in (Cui et al., 2011) Figs 1 and 2 in (Jacobsen et al., 2010), or Figs 1,2, 3 in (Sawamura et al., 2004). These bands are likely to correspond to a-secretase cleavage of different APP isoforms. In human brain cells, these isoforms range from 695 to 770 amino acids, based on alternative splicing of the Kunitz protease inhibitor domain (KPI) and MRC OX-2 antigen (OX-2) domains (Golde et al., 1990). The appearance/separation of these bands are depending on the gel conditions.

- The n of the experiments (n=4) was specified in the Figure legend even in our previous version.

Fig.6: this referee does not understand why authors used Aβ40 here! On the other hand, Aβ42 is used in panel b and c. This set of experiments should be done with Aβ42 that is more prone to aggregation.
In a more pathologically relevant context, authors should examine whether Trx80 expressing cells protect against toxicity harbored by aggregates prepared from AD-affected brains as in Walsh and Colleagues studies.

Response: Figure 6 aims to clarify the pro or anti- inflammatory effects of Trx80. We hypothesized that Trx80 could alter an inflammatory response, (as reported in the periphery) in an AD context. We used Abeta 40 and apoEs in the nitrite assay since these molecules were shown previously to have proinflammatory effects. In contrast, Abeta42 was previously reported do not affect nitrite production. This is now discussed in the manus where appropriate references are added. Nevertheless and following the advise of the referee, we show now Abeta 42± Trx80 effects. Text, figure legends and methodology have been changed to include this new results.

-We have now purified Abeta rich fractions from AD brains and analyzed their toxic effects (w or w/o Trx80) in NT and cells overexpresing Trx80 (Figure 6D). Methodological aspects and discussion of the data have been added to the paper.

Another important point is that authors do not directly establish whether α-secretase blockade by inhibitors increase Aβ-related toxicity in Trx1 expressing cells. This would reinforce the view that α-secretase-mediated Trx80 production could interfere with trx80 function. In this paper, although authors show that Trx80 derives from α-secretase cleavage of Trx1 and is apparently protecting when used as a recombinant protein or in cells overexpressing it, no direct link between the two events (i.e Trx1 cleavge by secretase) and protection against Ab toxicity is provided. Thus, authors should overexpress Trx1, assess the influence of this surexpression on Ab40/42-mediated toxicity and examine α-secretase inhibitors affect Trx1 specific phenotype.

Response: We think that this is a very important issue raised by the referee. We previously reported that overexpression of Trx1 protects against Abeta by inhibiting the ASK1 pathway (Akterin et al., 2006). Indeed a possible parallel increase of Trx80 in Trx1 overexpressing cells could contribute to the protective effect. We have now performed the experiments suggested by this referee, which are included in Figure 6. Accordingly the manus has been changed. We thank the reviewer for the suggestion as it has helped the interpretation of our data.

Minor points
-p7: Phorbol esters are known activators of α-secretase... Authors cite one of their reference that is not focusing on the point while original and many previous works are omitted!

Response: We have added the following the original reference for this point (Buxbaum et al., 1990).

REFEREE #2

Specific points:

- Trx1 plays a neuroprotective role because it is an antioxidant and an apoptosis inhibitor. The truncated form of Trx1, Trx80, lacks the neuroprotective role and it arises after cleaving of Trx1 by α secretases. Thus, it will be of interest to compare the levels of these α secretases (for example that of ADAM17) in AD and control samples (Western blot).

Response: In revising the manuscript, we have measured the levels of ADAM17 in Controls and AD brains (included as supplementary figure 3A). As previously reported, (Skovronsky et al., 2001) we found not changes in ADAM17 protein levels in AD compared to controls. It is also reported a loss of a -secretase activity in the disease (ie. (Tyler et al., 2002). Together with our data, this suggest that both a decrease in the precursor (trx1, (Akterin et al., 2006)) and a reduced a-secretase activity (Tyler et al., 2002) could be the cause of the decrease in Trx80 seen in AD. The revised discussion covers these considerations in more depth.

- In Figure 3a, a protein loading control is missing and Figure 3f lacks the parameters measured in the ordinate.

Response: We have corrected both concerns. Accordingly, Figure legend and text have been modified.

© European Molecular Biology Organization

- Also, some further editing is needed for the references of Vicent B and Checler F where volume and pages numbers are missing or for that of Zheng et al where the page numbers are not indicated.

Response: This was our typographical error, we have included the full references in the revised manuscript.

REFEREE #3

1) Data are generally quite interesting, covering human sample to protein level.
2) There have been no previous publications related to Trx80 in the field of clinical and basic neurobiology.
Therefore it is highly recommended to show comparative data between Trx80 and variety of anti-oxidant or redox-active principles in direct relation to alzheimer disease.

Response : Trx80 does not have a redox activity as Trx1 does. Our idea at this time is that overoxidation of Trx1 generates the substrate for cleavage to generate Trx80 (Hashemy and Holmgren, 2008). Indeed, oxidative stress is one of the components of AD pathology. However, in AD, there are less Trx1 levels and less enzymatic activity of the cutting enzyme (a-secretase). We have now expanded the introduction to include studies on Trx1 related molecules in AD. The following referees have been added (Cacho-Valadez et al., 2012; Kim et al., 2001)

3) Minor Comments
3-1 Figure 3B As the background level in this Figure is quite different from the data in oher panels, suitable careful correction is recommended.

Response : The fact is that in AD brains Trx80 Ir is almost absent (specially in cortex), including in the background. Trx80 is also secreted and it could be possible that a decrease in extracellular Trx80 could account for the differences. We prefer to do not modify digitally the pics, as they reflect the results using the same setup in the four panels. Another independent picture showing AD-cortex is shown in figure 3C.

3-2 Figure 5 This U937 Data using non-neuronal cell line cells should be supported by any suitable neuro_endocrine cell types.

Response: We do not understand the rational for using neuro-endocrine cells here. To our knowledge, there is not data suggesting that cells in the hypothalamus or the pituitary gland produce Trx80. In the revised form of the manus we have included confocal images in neuroblastoma cells which confirm the results seen on monocytes. These results are shown now in Supplementary figure 2D

3-3 Figure 6 Additional data is needed to show dose dependency.

Response: Three more concentrations of Trx80 have been analyzed (10nM, 100 nM and 1 mM) for their effects on Ab42 aggregation (Figure 6C). Only a short effect was found with 100nM that did not last for more than approx. 1 h. Trx80 (1 mM) showed similar anti-aggregant effects than Trx80 (5 mM). Noteworthy that the original concentration of Trx80 (5 mM) was effective already at low molar concentration ratio (1:5).

4) Suitable reference and discussion on the target molecules of thioredoxin family protein namely TBP2/TXNIP/VDUP1, Ref-1/APEX etc may improve the quality of this paper more relevant in the specific field of clinical and molecular neurodegenerative diseases.

Response: We have added a reference reviewing TBP2 in the introduction (Watanabe et al., 2010). The fundamental role of Trx1 and related proteins in cellular growth and survival, as well as in metabolic disorders and cancer, is underlined.

5) It is critically important to discuss the relationshhip between Trx80 and Trx-1, including the possibility of the reduction of Trx80 dimer by Trx-1.

Response: We have addressed this issue at the same time that responding to referee's #1 concern on Figure 1j. New experiments have been performed and are shown in a new Fif 1J. Combination of Trx80/Trx1 peptides, alone or incubated with cell do not change the aggregation pattern of those two molecules in SDS gels.

In summary, we have provided new and much more robust data for the major issue, which was to further investigate the link between Trx1 cleavage and protection against Abeta toxicity. We have also added, where requested, additional experiments and clarified experimental approaches such as the effects of Trx1 on Trx80 aggregation or the levels of ADAM 17 in AD brains. Finally, we have gone through the manuscript and corrected minor requests and the typographical errors and missed references pointed out by the referees. We hope that this revised version of our manuscript is suitable for publication in EMM.

REFERENCES

Akterin S, Cowburn RF, Miranda-Vizuete A, Jimenez A, Bogdanovic N, Winblad B and Cedazo-Minguez A. (2006) Involvement of glutaredoxin-1 and thioredoxin-1 in beta-amyloid toxicity and Alzheimer's disease. Cell Death Differ, 13, 1454-1465.

Buxbaum JD, Gandy SE, Cicchetti P, Ehrlich ME, Czernik AJ, Fracasso RP, Ramabhadran TV, Unterbeck AJ and Greengard P. (1990) Processing of Alzheimer beta/A4 amyloid precursor protein: modulation by agents that regulate protein phosphorylation. Proc Natl Acad Sci U S A, 87, 6003-6006.

Buxbaum JD, Liu KN, Luo Y, Slack JL, Stocking KL, Peschon JJ, Johnson RS, Castner BJ, Cerretti DP and Black RA. (1998) Evidence that tumor necrosis factor alpha converting enzyme is involved in regulated alpha-secretase cleavage of the Alzheimer amyloid protein precursor. J Biol Chem, 273, 27765-27767.

Cacho-Valadez B, Munoz-Lobato F, Pedrajas JR, Cabello J, Fierro-Gonzalez JC, Navas P, Swoboda P, Link CD and Miranda-Vizuete A. (2012) The characterization of the Caenorhabditis elegans mitochondrial thioredoxin system uncovers an unexpected protective role of thioredoxin reductase 2 in beta-amyloid peptide toxicity. Antioxid Redox Signal, 16, 1384-1400.

Cui H, Hung AC, Klaver DW, Suzuki T, Freeman C, Narkowicz C, Jacobson GA and Small DH. (2011) Effects of heparin and enoxaparin on APP processing and Abeta production in primary cortical neurons from Tg2576 mice. PLoS One, 6, e23007.

Golde TE, Estus S, Usiak M, Younkin LH and Younkin SG. (1990) Expression of beta amyloid protein precursor mRNAs: recognition of a novel alternatively spliced form and quantitation in Alzheimer's disease using PCR. Neuron, 4, 253-267.

Hartmann D, de Strooper B, Serneels L, Craessaerts K, Herreman A, Annaert W, Umans L, Lubke T, Lena Illert A, von Figura K and Saftig P. (2002) The disintegrin/metalloprotease ADAM 10 is essential for Notch signalling but not for alpha-secretase activity in fibroblasts. Hum Mol Genet, 11, 2615-2624.

Hashemy SI and Holmgren A. (2008) Regulation of the catalytic activity and structure of human thioredoxin 1 via oxidation and S-nitrosylation of cysteine residues. J Biol Chem, 283, 21890-21898.

Jacobsen KT, Adlerz L, Multhaup G and Iverfeldt K. (2010) Insulin-like growth factor-1 (IGF-1)-induced processing of amyloid-beta precursor protein (APP) and APP-like protein 2 is mediated by different metalloproteinases. J Biol Chem, 285, 10223-10231.

Kim SH, Fountoulakis M, Cairns N and Lubec G. (2001) Protein levels of human peroxiredoxin subtypes in brains of patients with Alzheimer's disease and Down syndrome. J Neural Transm Suppl, 223-235.

Sawamura N, Ko M, Yu W, Zou K, Hanada K, Suzuki T, Gong JS, Yanagisawa K and Michikawa M. (2004) Modulation of amyloid precursor protein cleavage by cellular sphingolipids. J Biol Chem, 279, 11984-11991.

Skovronsky DM, Fath S, Lee VM and Milla ME. (2001) Neuronal localization of the TNFalpha converting enzyme (TACE) in brain tissue and its correlation to amyloid plaques. J Neurobiol, 49, 40-46.

Tyler SJ, Dawbarn D, Wilcock GK and Allen SJ. (2002) alpha- and beta-secretase: profound changes in Alzheimer's disease. Biochem Biophys Res Commun, 299, 373-376.
Watanabe R, Nakamura H, Masutani H and Yodoi J. (2010) Anti-oxidative, anti-cancer and anti-inflammatory actions by thioredoxin 1 and thioredoxin-binding protein-2. Pharmacol Ther, 127, 261-270.

2nd Editorial Decision 06 July 2012

Thank you for the submission of your revised manuscript to EMBO Molecular Medicine. We have now received the enclosed reports from the referees that were asked to re-assess it. As you will see the reviewers are now globally supportive and I am pleased to inform you that we will be able to accept your manuscript pending the following final amendments:

- Some parts of the Material and Methods in the main manuscript text seem redundant with the respective parts in the Supplementary Information. Could you please check this and remove potential redundancies?

- Please address the minor concern raised by Reviewer #1.

Please submit your revised manuscript within two weeks. I look forward to seeing a revised form of your manuscript as soon as possible.

Yours sincerely,

Editor
EMBO Molecular Medicine

***** Reviewer's comments *****

Referee #1:

The authors adequately adressed my concerns and improved significantly their article. Even if I was referee #1, looking at referee's #2 report and author's reply, unless I am wrong, the references Vincent and Checler and Zheng et al. have not been updated!

Referee #2:

Upon revision, this is an improved manuscript that has taken into account all the points raised by this reviewer.

2nd Revision - authors' response 14 July 2012

We are delighted to see that the editorial board are able to accept our revised manuscript "Thioredoxin-80 is a product of alpha-secretase cleavage that inhibits amyloid-beta aggregation and is decreased in Alzheimer's disease brain" for publication in *EMBO Molecular Medicine*. Once again, we thank the reviewers for appreciating our results and for their most valuable comments. We have now removed redundancies in the Supplementary Information as suggested and corrected the minor concerns raised by Reviewer # 1.
