# Supplementary material for: Thioredoxin-80 is a product of alpha-secretase cleavage that inhibits amyloid-beta aggregation and is decreased in Alzheimer's disease brain
Source: EMBO Mol Med. 2012 Aug 30;4(10):1097–111. doi: 10.1002/emmm.201201462 (PMC3491839; doi:10.1002/emmm.201201462)
Supplement: Supplementary file 2 [file emmm0004-1097-SD2.pdf]

Thioredoxin-80 is a product of alpha-secretase cleavage that inhibits amyloid-beta aggregation and is decreased in Alzheimer's disease brain.

Francisco Gil-Bea et al.

SUPPLEMENTARY INFORMATION

TABLE OF CONTENTS

1. Legends to supplementary data page 2
2. Supplementary Figure 1 page 4
3. Supplementary Figure 2 page 5
4. Supplementary Figure 3 page 6
5. Experimental procedures (extended) page 7

1. LEGENDS TO SUPPLEMENTARY DATA

Supplementary Figure 1

Entire gel scans (photo scans or CCD camera images) for Figures 1E **(A)**, 1H (**B**), 1I (**C**) and 3A(**D**).

In figure **Suppl. 1B**: lanes 1,2,3: Non-transfected cells; 4,5,6: Trx80 transfected cells; 7,8,9: Trx1 transfected cells. Each lane contain extracts of a different clone. Inmunoblottings for Trx80 and actin are shown.

In figure **Suppl. 1C**: lane 1: Non-transfected cells in serum-free media; 2: Non-transfected cells in serum-containing media; 3: Control cells (Darmafect treated); 4 Scramble RNA (scRNA)-treated cells; 5 and 6 siRNA of Trx1 (12.5 and 25 nM respectively). Two different experiments are shown in immnunoblots for Trx1, Trx80 and actin.

Supplementary Figure 2

A. Trx80 immunorreactivy in hippocampal sections of Control and AD brains (lower magnification than Figure 3b)**;** SH-SY5Y neuroblastoma cells; and U937 cells untreated of treated with PMA (as described in the article).

B. Inmunofluorescence of Trx80 (green) in SH-SY5Y cells and U937 monocytes. Phorbol 12-myristate 13-acetate (PMA) (50ng/ml) increased intracellular levels of Trx80. Trx80 immunoreactivity showed a dotted staining pattern

C. PMA–mediated increased of Trx80 levels in media U937 cells were not affected by serum free conditions. Treatment was for 24 or 48 h with 50 ng/mL PMA. (+) indicates 10% FCS containing media and (-) indicates serum free conditions.

D. Co-localization of Trx1 (green) and ADAM17 (red) in untreated SH-SY5Y cells. Both single plane and z-stack images are shown. The co-staining (yellow) of Trx1 and ADAM17 was present in the cytosol. As seen in U937 cells (Figure 5), the dotted pattern suggests that Trx1 and ADAM17 could co-localize in vesicular compartments in the cytoplasm. DAPI is used to stain nuclei (blue).

Supplementary Figure 3

A. Nitric oxide production measured in mixed glial rat primary cultures as in Figure 6b but using 100nM Trx80. LPS = lipopolysaccharide (90ng/mL); C = untreated cells; Aβ = Aβ(1-40) (10μM); E3 = apoE3 (10nM); E4 = apoE4 (10nM). Data are expressed as ng of nitrites produced per μg of total protein. Bars represent mean values of three experiments ± SEM.

B. Immunoblotting of human cortical brain samples revealed no changes in ADAM17 protein levels between controls (C) and AD. Ponceau staining is shown as loading control.

Supplementary Figure 1

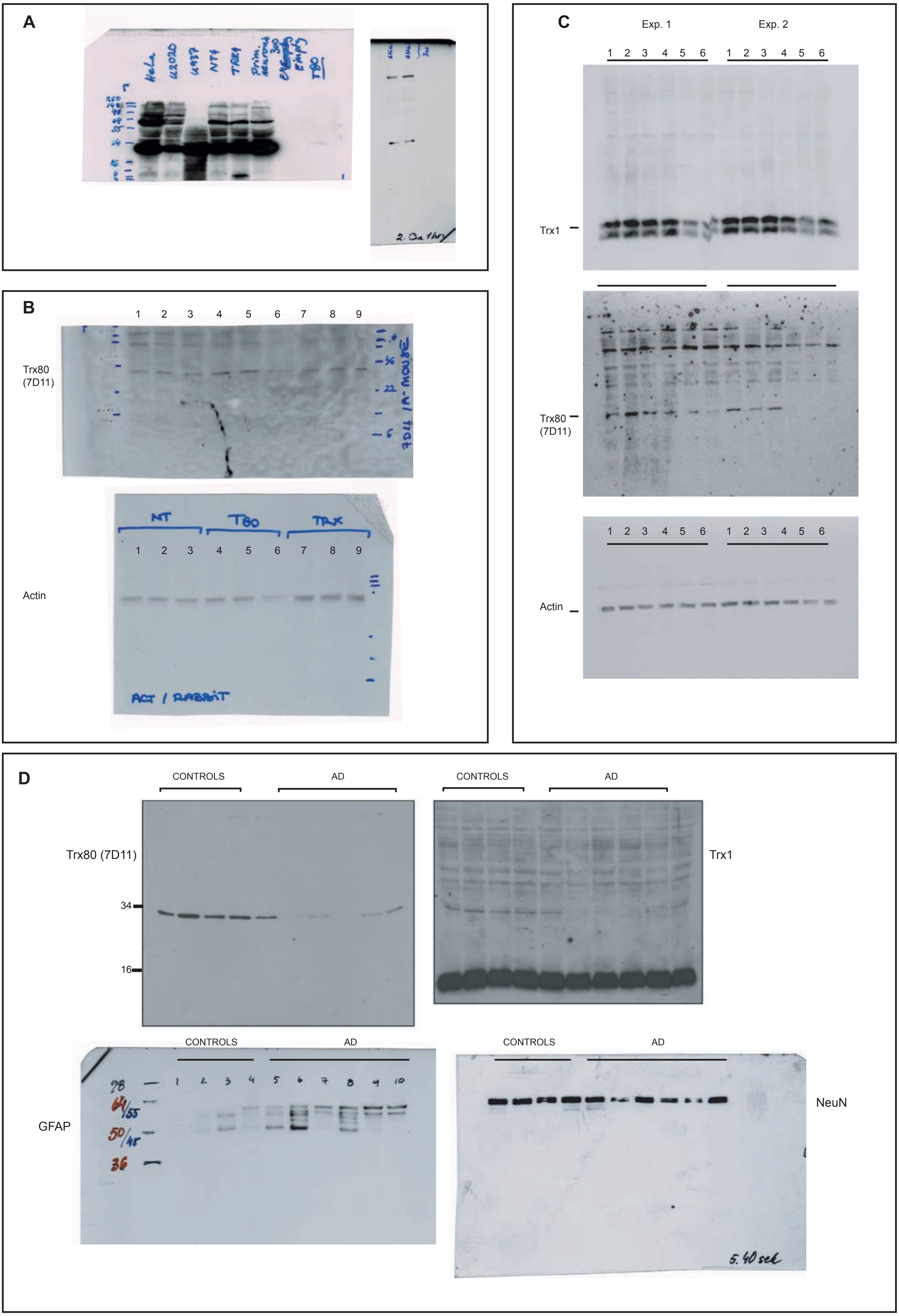

Supplementary Figure 2

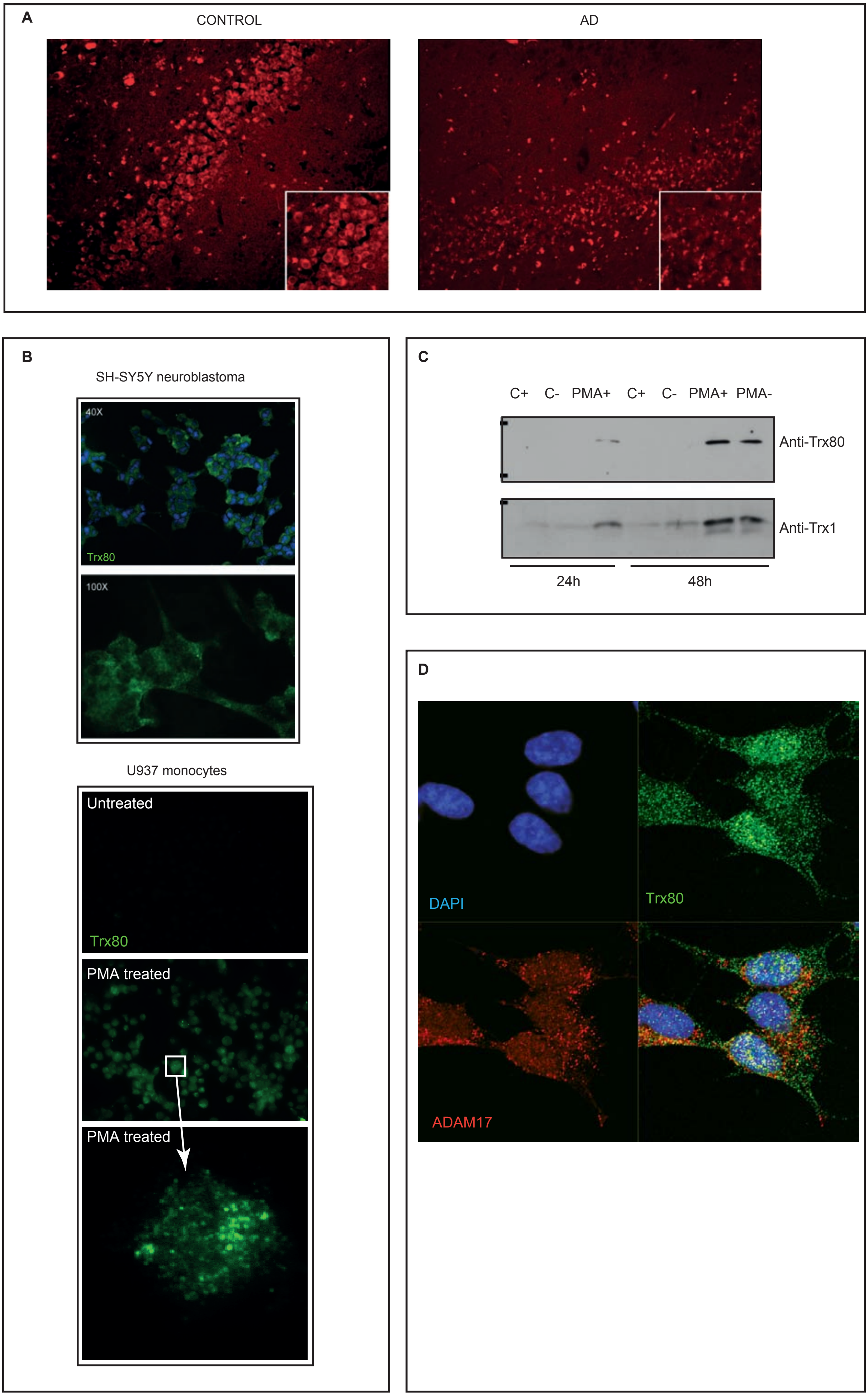

Supplementary Figure 3

A

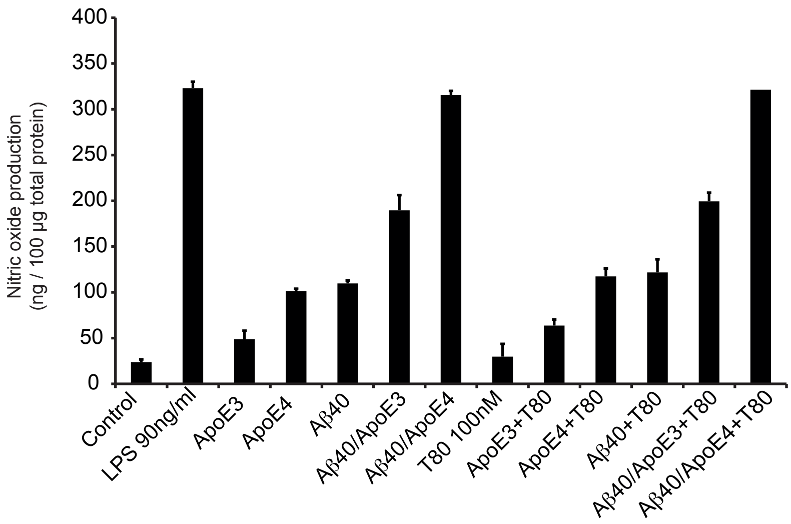

B

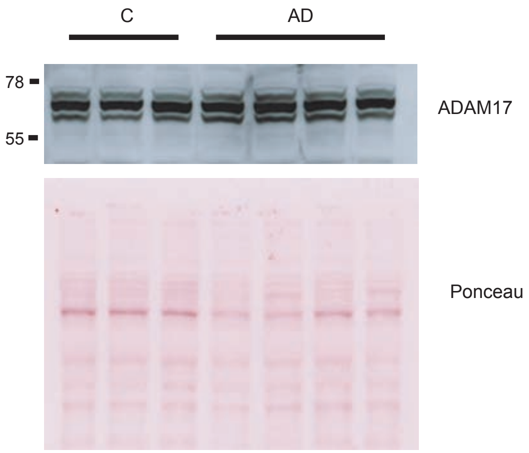

5. EXPERIMENTAL PROCEDURES (EXTENDED)

Brain tissue preparation

For immunohistochemistry the tissue was fixed in buffered 4% formaldehyde and embedded in paraffin. For immunoblotting brain samples were sonicated 4 x 15 seconds in TE-buffer (50 mM Tris-HCl, pH 7.4, 1 mM EDTA) with freshly added protease inhibitor cocktail (Sigma-Aldrich, Saint Louis, MO, USA, 1:500) and phosphatase inhibitors (20 mM β-glycerophosphate, 2nM okadaic acid, 50 mM sodium flouride, 1 mM sodium orthovanadate). After sonication the samples were centrifuged at 10 000 x g for 10 min and 100 000×g for 20 min at +4°C.

Immunohistochemical analysis

Sections (7 μm-thick) were mounted onto Superfrost plus-glass (Menzel Braunschweig, Germany), baked at 37°C overnight, dewaxed and hydrated. Then sections were subjected to microwave antigen retrieval in sodium citrate buffer (10 mM, pH 6) at 700 W for 10 min and blocked for non-specific sites by addition of 10% of serum (from the species in which the secondary antibody was produced) and 10% of BSA in PBS with 0.3% of Triton X-100 (PBS-Tx) for 30 min prior to incubations with the primary antibody (7D11 against Trx80 was from IMCO Corporation Ltd AB, Sweden; Anti-Aβ42 abs were fSections were then incubated for 2h with secondary antibody (see Table 1) in PBS-Tx with 0.2% serum and 1% BSA, after which counterstaining with DAPI was performed. Finally the sections were rinsed in PBS-Tx and mounted in fluorescence mounting medium (DAKO Cytomation, Glostrup, Denmark). The sections were thoroughly washed in PBS-Tx between different steps. The protocol for antibodies was repeated to assure the reproducibility of results. All sections were treated simultaneously under the same conditions. For control staining the primary antibody was omitted. Nikon Eclipse E800 microscope and Hamamatsu ORCA-ER digital camera were used for capturing images from the immunohistochemistry.

Human primary cultures

The glial cultures consisted almost entirely of astrocytes since at this embryonal age very few cells of myeloid origin have migrated into the brain. Cells were seeded

separately on cover slips (20 000 cells/cm^2), pre-coated with poly-D-lysine (Sigma-Aldrich, St. Louis, MO, USA) in PBS.

Preparation of cell lysate and media for immunoblotting

U937 cells are non-adherent, but some treatments will differentiate them into adherent macrophage-like cells. Thus for U937, after treatment, media and floating cells were collected and separated by centrifugation (1000×g for 10 min) at +4°C. Attached cells were rinsed in PBS and harvested in PBS by centrifugation at +4°C (1500×g for 5 min). For adherent cell lines and primary cultures media was collected and centrifuged (1000 x g for 10 min) at +4°C. The resulting pellet was discarded. Cells were rinsed in PBS and harvested in PBS by centrifugation at +4°C (1500×g for 5 min). All cell types were lysed by applying lysis buffer (50 mM Tris-HCl pH 7.5, 150 mM NaCl, 1 % Triton X-100, 2 mM EDTA, 2 mM EGTA), with protease inhibitor cocktail (Sigma-Aldrich, Saint Louis, MO, USA) at dilution 1:500 added freshly, to the pellet and incubated 30-60 min on ice before centrifugation (15 000 x g for 10 min) at +4°C. Any cell pellet from the U937 media was lysed in the same fashion and later pooled with the lysate from the attached cells.

Trx80 measurements by specific sandwich ELISA.

Standard samples of recombinant Trx80 (IMCO Corporation Ltd AB, Sweden) were aliquoted in 10 µl at 50 µg/ml in PBS with 0.5% bovine serum albumin and kept at -70 °C. 96-well plates were coated with 50µl of 15µg/ml anti-Trx80 monoclonal mouse antibody (clone 7D11) (IMCO Corporation Ltd AB, Sweden) in PBS and incubated at 4°C O/N. Unspecific protein binding sites were blocked by incubation with 200µl of blocking buffer (0.5% BSA, 0.05% Tween 20, 0.02% NaN3 in PBS) 2h in RT. Plates were washed four times with washing buffer (0.05% Tween 20 in PBS). Standard dilutions of Trx80 (0.2 ng/ml - 100 ng/ml) were prepared in blocking buffer, while CSF samples were undiluted. Fifty µl of standards or samples were added in duplicates and incubated O/N. Thereafter, plates were washed four times with washing buffer prior to incubation for 2h with biotinylated goat anti-Trx1 polyclonal antibody (IMCO Corporation Ltd AB, Sweden) (2 µg/ml in 50 µl, at RT). Plates were washed four times with washing buffer prior addition to 50 µl of alkaline phosphatase-conjugated streptavidin (1:1000, 1h, RT, in blocking buffer). Plates were

then washed six times in washing buffer and 50 μl of 1 mg/ml p-nitrophenyl phosphate dissolved in 10% diethanolamine, 0.02% NaN_3, 0.5 mM $MgCl_2$, pH 9.8 was added to each well. Absorbance was recorded at 405nm.
